# Single-cell RNA sequencing reveals distinct tumor microenvironmental patterns in lung adenocarcinoma

**DOI:** 10.1038/s41388-021-02054-3

**Published:** 2021-10-18

**Authors:** Philip Bischoff, Alexandra Trinks, Benedikt Obermayer, Jan Patrick Pett, Jennifer Wiederspahn, Florian Uhlitz, Xizi Liang, Annika Lehmann, Philipp Jurmeister, Aron Elsner, Tomasz Dziodzio, Jens-Carsten Rückert, Jens Neudecker, Christine Falk, Dieter Beule, Christine Sers, Markus Morkel, David Horst, Nils Blüthgen, Frederick Klauschen

**Affiliations:** 1 Institute of Pathology, Charité—Universitätsmedizin Berlin, corporate member of Freie Universität Berlin and Humboldt-Universität zu Berlin, Berlin, Germany; 2 Berlin Institute of Health, Charité—Universitätsmedizin Berlin, Berlin, Germany; 3 Core Unit Bioinformatics (CUBI), Berlin Institute of Health at Charité—Universitätsmedizin Berlin, Berlin, Germany; 4 IRI Life Sciences, Humboldt University of Berlin, Berlin, Germany; 5 German Cancer Consortium (DKTK) Partner Site Berlin, German Cancer Research Center (DKFZ), Heidelberg, Germany; 6 Department of Surgery, Campus Charité Mitte and Campus Virchow-Klinikum, Charité—Universitätsmedizin Berlin, corporate member of Freie Universität Berlin and Humboldt-Universität zu Berlin, Berlin, Germany; 7 Institute of Transplant Immunology, Hannover Medical School, Hannover, Germany; 8 TTU-IICH Hannover-Braunschweig Site, German Center for Infectious Diseases (DZIF), Braunschweig, Germany

**Keywords:** Sequencing, Non-small-cell lung cancer, Tumour heterogeneity

## Abstract

Recent developments in immuno-oncology demonstrate that not only cancer cells, but also the tumor microenvironment can guide precision medicine. A comprehensive and in-depth characterization of the tumor microenvironment is challenging since its cell populations are diverse and can be important even if scarce. To identify clinically relevant microenvironmental and cancer features, we applied single-cell RNA sequencing to ten human lung adenocarcinomas and ten normal control tissues. Our analyses revealed heterogeneous carcinoma cell transcriptomes reflecting histological grade and oncogenic pathway activities, and two distinct microenvironmental patterns. The immune-activated CP²E microenvironment was composed of cancer-associated myofibroblasts, proinflammatory monocyte-derived macrophages, plasmacytoid dendritic cells and exhausted CD8+ T cells, and was prognostically unfavorable. In contrast, the inert N³MC microenvironment was characterized by normal-like myofibroblasts, non-inflammatory monocyte-derived macrophages, NK cells, myeloid dendritic cells and conventional T cells, and was associated with a favorable prognosis. Microenvironmental marker genes and signatures identified in single-cell profiles had progonostic value in bulk tumor profiles. In summary, single-cell RNA profiling of lung adenocarcinoma provides additional prognostic information based on the microenvironment, and may help to predict therapy response and to reveal possible target cell populations for future therapeutic approaches.

## Introduction

Lung cancer has a poor prognosis and accounts for the majority of new cases and deaths of cancer worldwide [1]. The most common subtype of lung cancer, in particular in nonsmokers, is lung adenocarcinoma [2]. Presently, molecular profiling of driver mutations is guiding treatment with targeted therapies [3, 4] and expression of PD-L1 in tumor cells is used to predict response to immune checkpoint inhibitors [5]. Comprehensive characterization including features of the tumor microenvironment could provide more precise patient stratifications.

Cancers are multicellular communities comprising malignant epithelial cells and different types of nonmalignant immune and stromal cells which exhibit dynamic and reciprocal interactions. Modulation of immune responses, remodeling of the extracellular matrix and neoangionesis essentially determine the aggressiveness of cancer [6]. Current bulk omics analyses do not allow high-resolution characterization of cellular diversity of the tumor microenvironment. However, comprehensive single-cell profiling of patient tissue is emerging as an essential tool to estimate the clinical relevance of individual cell types in the tumor.

In this study, we analyzed tumor epithelial cells and associated nonmalignant cells of the tumor microenvironment of lung adenocarcinomas by single-cell RNA sequencing. Extending previous single-cell studies [7–10], we found that the heterogeneous cellular composition of the tumor microenvironment across patients follows specific patterns that were associated with the differentiation grade of carcinoma cells. Translation of our findings to an retrospective cohort characterized by bulk gene expression revealed potential prognostic relevance of microenvironmental patterns. We conclude that a comprehensive profiling of the lung adenocarcinoma microenvironment may help to reveal novel clinically relevant tumor subtypes based on carcinoma cells and microenvironmental features.

## Results

### Single-cell RNA sequencing uncovers the cellular diversity of lung adenocarcinomas

**Fig. 1:**
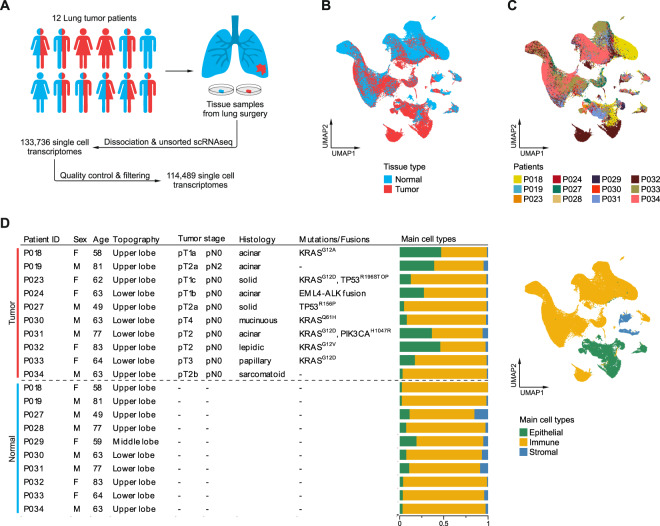
Single-cell RNA sequencing of lung adenocarcinomas. **A** Schematic representation of the workflow, ten normal (blue) and ten tumor (red) tissue samples were obtained from 12 patients. **B**, **C** UMAPs based on the top 15 principal components of all single-cell transcriptomes after filtering, color-coded by (**B**) tissue type, or (**C**) patient. **D** Overview of clinical features, clinically relevant oncogenic mutations and gene fusions; quantification of main cell types per patient and UMAP of all single-cell transcriptomes color-coded by main cell type.

To study the cellular composition of lung adenocarcinoma, ten normal lung and ten lung adenocarcinoma fresh tissue samples were collected during surgery and subjected to unsorted single-cell RNA sequencing, yielding 114,489 high-quality transcriptomes after quality control and filtering (Fig. 1A, Supplementary Fig. 1A, B). Evaluation of consecutive H&E stained tissue sections showed tumor morphology ranging from well differentiated lepidic to poorly differentiated sarcomatoid growth patterns (Supplementary Fig. 2).

Analysis and visualization by Uniform Manifold Approximation and Projection (UMAP) [11] showed that single-cell transcriptomes of different tissue types or patients intermingled in many clusters, excluding general batch effects, and partly formed tumor- or patient-specific clusters, indicating underlying biological differences (Fig. 1B, C, Supplementary Fig. 1C). To uncover which cellular compartments account most for interpatient variability, we analyzed single-cell transcriptomes for the expression of epithelial, immune, and stromal marker genes. In total, 20,450 epithelial, 89,766 immune, and 4273 stromal single-cell transcriptomes were covered, suggesting an overrepresentation of immune cell transcriptomes as observed in other studies [9, 12]. Yield of epithelial transcriptomes varied by histological subtype, as we observed a frequency <10% in solid/sarcomatoid, but up to >40% in lepidic/acinar carcinomas (Fig. 1D). Epithelial cells showed the highest degrees of interpatient heterogeneity (Fig. 1C, D).

### Intertumoral heterogeneity of tumor epithelial cells reflects differentiation gradients

**Fig. 2:**
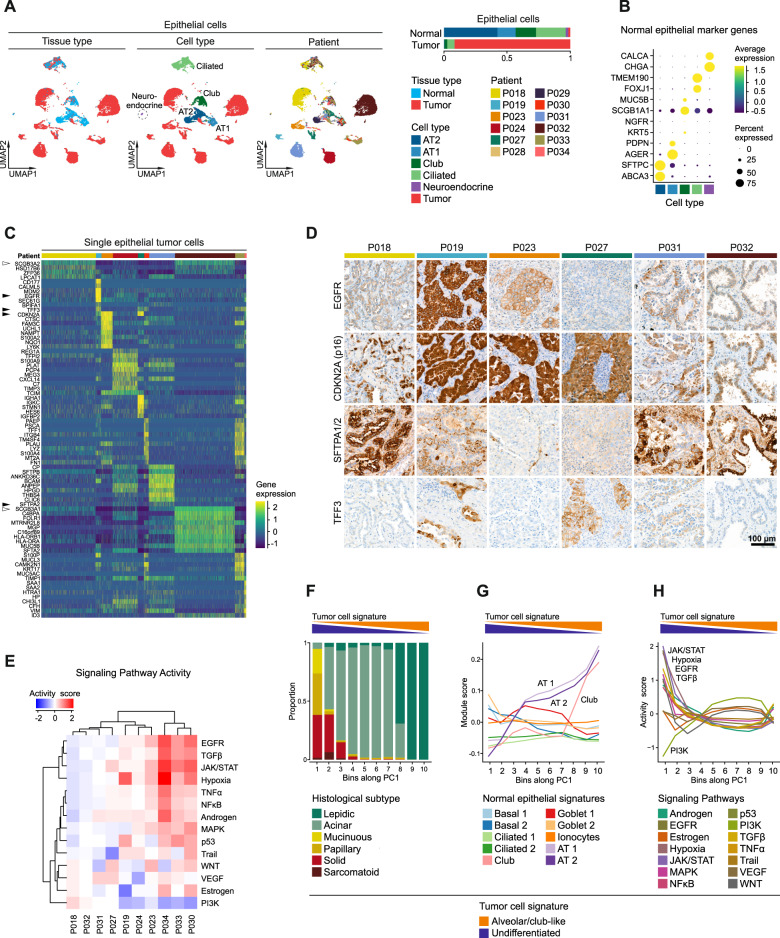
Intertumoral heterogeneity of tumor epithelial cells in lung adenocarcinomas. **A** UMAPs based on the top 20 principal components of all epithelial single-cell transcriptomes color-coded by tissue type, cell type and patient, and quantification of epithelial cell types per tissue type, AT1, alveolar type 1 cells, AT2, alveolar type 2 cells. **B** Average gene expression of selected marker genes for normal epithelial cell types. **C** Differentially expressed genes in tumor epithelial cells grouped by patients, maximum top ten genes showed per patient, for patient color code see (**A**). **D** Immunohistochemical staining of proteins encoded by selected differentially expressed genes indicated by black arrowheads in (**C**). **E** Mean pathway activity scores of tumor epithelial cells grouped by patient. **F** Distribution of histological subtypes, (**G**) mean module scores of normal epithelial cell type gene signatures, and (**H**) mean pathway activity scores of tumor epithelial cells sorted along principal component 1 (PC1). **F**, **G**, **H** Principal component analysis based on gene expression of all tumor epithelial single-cell transcriptomes; schematic depiction of tumor cell signature module scores along PC1.

To further dissect interpatient variability within the epithelial cell compartment, epithelial transcriptomes were subset and reclustered (Supplementary Fig. 3A). Clusters were defined as normal or tumor cell clusters, based on tissue origin (Supplementary Fig. 3B), which was largely congruent with the copy-number status of cells (Supplementary Fig. 4A, B), and demonstrated a tumor purity >90%. Within the normal cell clusters, we found alveolar type 1 and 2, club, ciliated, and even a small cluster of neuroendocrine cells (Fig. 2A), which were characterized by expression of typical individual marker genes (Fig. 2B) and gene signatures (Supplementary Fig. 5A, B) [13, 14]. The club cell cluster also expressed basal cell marker genes such as *NGFR* and *KRT5* indicating an admixture of small amounts of basal cells (Fig. 2B). Tumor cell clusters segregated from normal cell clusters and were mainly patient-specific, indicating intertumoral heterogeneity (Fig. 2A). This was underlined by a variety of genes differentially expressed across tumors such as *EGFR, TFF3, CDKN2A*, and *SFTPA2* (Fig. 2C, black arrowheads), correlating with protein expression as shown by immunostaining (Fig. 2D). We quantified oncogenic signal strengths by pathway target gene signature expression and found highly variable activities for EGFR, TGFβ, JAK/STAT, Hypoxia, and PI3K signaling across the different patients (Fig. 2E). These signal strengths were largely unrelated to the mitotic activity of tumor epithelial cells (Supplementary Fig. 3C). p53 signaling was significantly reduced in tumors harboring *TP53* mutations, whereas pathway activity scores for EGFR and MAPK signaling were not significantly higher in *KRAS*-mutated compared to *KRAS*-wildtype tumors (Supplementary Fig. 3D).

Despite obvious intertumoral heterogeneity, we also noted shared features of epithelial tumor cell transcriptomes across patient subgroups. In order to emphasize similarities between tumors, epithelial transcriptomes were embedded in low-dimensional UMAPs (4, 6, 8 instead of 20 dimensions). Here, tumor cells clustered by histological subtype rather than by patient (Supplementary Fig. 3E), and the first principal component (PC1) displayed a gradient along histological grades (Fig. 2F, Supplementary Fig. 3E, F). Interestingly, *SCGB3A1* and *SCGB3A2* (Fig. 2C, white arrowheads), two genes that were previously associated with lung development [15], were positively correlated with PC1 (Supplementary Fig. 3G, arrowheads). Moreover, gene signature scores of normal lung cell types [13] along PC1 showed a strong positive correlation with gene expression profiles of alveolar type 1 and 2 as well as club cells (Fig. 2G). Together, this indicates that PC1 reflects the degree of differentiation of tumor epithelial cells. Hence, the top 30 genes positively and negatively correlated with PC1 were defined as an “alveolar/club-like” and “undifferentiated” tumor cell signature, respectively (Fig. 2F–H, Supplementary Fig. 3G, H). While tumor cells with different degrees of differentiation exhibited no clear differences in mitotic activity (Supplementary Fig. 3I), we found high pathway activity scores for JAK/STAT, Hypoxia, EGFR and TGFβ signaling in “undifferentiated”, and high scores for PI3K signaling in “alveolar/club cell-like” tumor epithelial cells, respectively (Fig. 2H). We conclude that tumor epithelial cells of different lung adenocarcinoma patients exhibit transcriptional patterns along a spectrum ranging from undifferentiated to alveolar/club cell-like phenotypes correlating with distinct oncogenic pathway activity.

### Two subtypes of myofibroblasts constitute the tumor stromal microenvironment

**Fig. 3:**
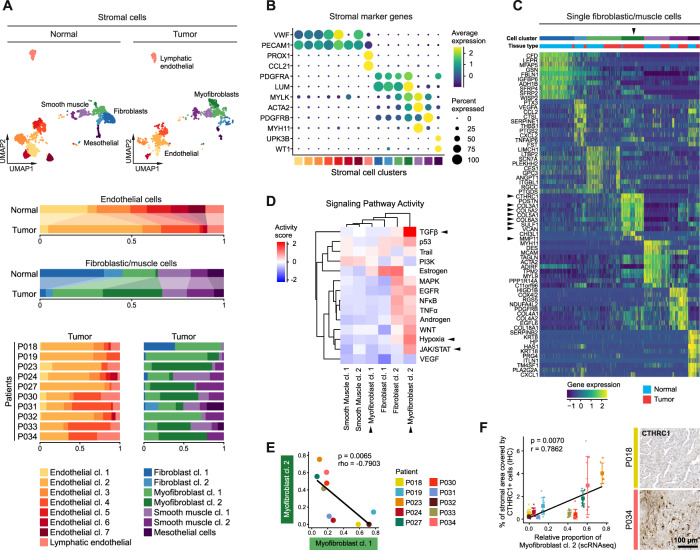
Composition of the stromal microenvironment of lung adenocarcinomas. **A** UMAPs based on the top 20 principal components of all stromal single-cell transcriptomes split by tissue type, color-coded by cell cluster; and relative quantification of endothelial and fibroblastic/muscle cell clusters per tissue type and, for tumor samples, per patient. **B** Average gene expression of selected marker genes for stromal cell clusters, for cell cluster color code see (**A**). **C** Differentially expressed genes of fibroblastic/muscle cell clusters, maximum top ten genes showed per cell cluster, for cell cluster color code see (**A**), black arrowheads indicate relevant marker genes of myofibroblast cluster 2 mentioned in the main text. **D** Mean pathway activity scores of different fibroblastic/muscle cell clusters, mesothelial cells excluded, black arrowheads indicate relevant pathways of myofibroblast clusters 1 and 2 mentioned in the main text. **E** Correlation of the relative quantity of myofibroblast clusters 1 and 2, color-coded by patient; Spearman’s correlation statistics, linear regression line. **F** Immunohistochemical staining of CTHRC1 as marker for myofibroblast cluster 2 (see also (**C**)), quantification of proportion of stromal areal covered by CTHRC1+ cells, mean ± s.d., *n* = 10 per patient, for patient color code see (**E**); Pearson’s correlation statistics and linear regression line using mean values per patient.

We subset and analyzed stromal cells from both normal and tumor tissue samples. Different clusters of endothelial and lymphatic endothelial cells, fibroblasts, myofibroblasts and smooth muscle cells and mesothelial cells (Fig. 3A, Supplementary Fig. 6A) were identified by marker genes (Fig. 3B) and gene signatures (Supplementary Fig. 5A, B) [13, 14]. Tumor endothelial cells were mainly represented by clusters 2 and 4 (Fig. 3A), and showed high expression of angiogenesis markers such as *VWA1* and *HSPG2*, as well as *INSR*, encoding an endothelial marker protein and possible therapeutic target [9] (Supplementary Fig. 6B, arrowheads).

Among the fibroblastic/muscle cell clusters, we detected a shift from fibroblast to myofibroblast cell clusters in tumor tissues (Fig. 3A), which we also observed in an independent dataset (Supplementary Fig. 7A). Myofibroblast clusters were characterized by expression of both fibroblastic marker genes, such as *PDGFRA* and *LUM*, and smooth muscle marker genes, such as *MYLK* and *ACTA2* (Fig. 3B). Notably, myofibroblast cluster 2 was almost exclusively found in tumor tissues while myofibroblast cluster 1 encompassed normal and tumor tissues. Myofibroblast cluster 2 displayed high expression of collagens such as *COL3A1*, *COL5A1, COL5A2* and *COL6A3*, other matrix proteins such as *VCAN*, as well as matrix-degrading enzymes such as *SULF1* and *MMP11*, suggesting roles in extracellular matrix remodeling (Fig. 3C, arrowheads). Myofibroblast cluster 2 was also characterized by high activity of TGFβ and JAK/STAT signaling as well as hypoxia-induced pathways (Fig. 3D), which are known features of cancer-associated myofibroblasts [16, 17]. In contrast, myofibroblast cluster 1 exhibited low activities of these pathways. Relative proportions of myofibroblast clusters 1 and 2 within the fibroblastic/muscle cell compartment correlated inversely across patients (Fig. 3E). The distribution of myofibroblast cluster 2 cells could be validated by immunostaining for the cluster-specific marker CTHRC1 (Fig. 3F, see also Fig. 3C). We conclude that myofibroblasts cluster 1 and 2 represent “normal-like” and “cancer-associated” phenotypes of myofibroblasts, respectively, and either of them can predominate the stromal microenvironment.

### The tumor immune microenvironment exhibits pro- and non-inflammatory traits

**Fig. 4:**
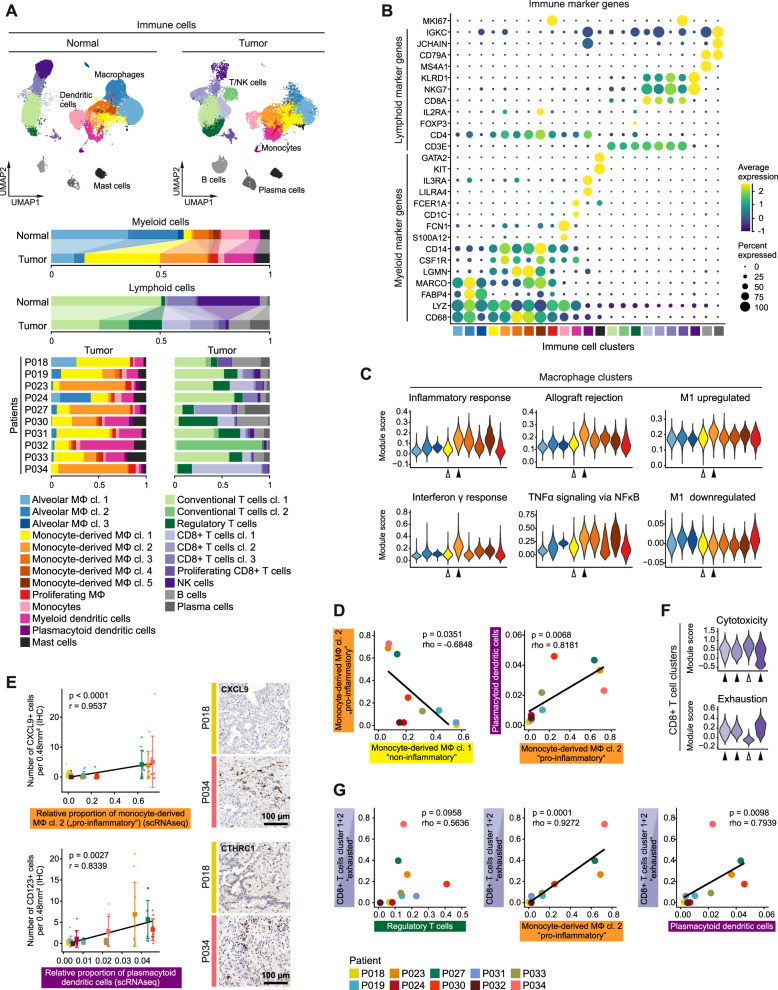
Composition of the immune microenvironment of lung adenocarcinomas. **A** UMAPs based on the top 20 principal components of all immune single-cell transcriptomes split by tissue type, color-coded by cell cluster; and relative quantification of myeloid and lymphoid cell clusters per tissue type and, for tumor samples, per patient. **B** Average gene expression of selected marker genes for immune cell clusters, for cell cluster color code see (**A**). **C** Module scores of gene signatures related to inflammation and M1/M2 polarization of different macrophage clusters, white and black arrowheads indicate monocyte-derived macrophage clusters 1 and 2, respectively, for cell cluster color code see (**A**). **D** Correlation of the relative quantity of selected myeloid immune cell clusters, for patient color code see (**G**); Spearman’s correlation statistics, linear regression line. **E** Immunohistochemical staining of CXCL9 and CD123 as markers for monocyte-derived macrophage cluster 2 and plasmacytoid dendritic cells, respectively, quantification of CXCL9+ or CD123+ cells per 0.48 mm², mean ± s.d., *n* = 10 per patient, for patient color code see (**G**); Pearson’s correlation statistics and linear regression line using mean values per patient. **F** Module scores of gene signatures related to cytotoxicity and exhaustion of different CD8+ T cell clusters, white and black arrowheads indicate cell clusters enriched in normal or tumor tissue, respectively, for cell cluster color code see (**A**). **G** Correlation of the relative quantity of selected lymphoid and myeloid immune cell clusters, color-coded by patient; Spearman’s correlation statistics, linear regression line.

We next subset and analyzed immune cells of the tumor microenvironment. We identified different clusters of tissue-resident and monocyte-derived macrophages, monocytes, myeloid and plasmacytoid dendritic cells, mast cells, and T, NK, B, and plasma cells (Fig. 4A, Supplementary Fig. 8A–C) based on typical marker genes (Fig. 4B) and gene signatures (Supplementary Fig. 5A, B) [13, 14].

Although immune cell clusters usually encompassed both normal and tumor tissue-derived transcriptomes, we noted quantitative shifts in the cellular composition of the tumor immune microenvironment (Fig. 4A). Among myeloid cell types in the tumor, monocyte-derived macrophages, defined by markers such as *CD14, CSF1R* and *LGMN* (Fig. 4B) [18, 19] and dendritic cells were increased, while tissue-resident macrophages and monocytes were decreased, which we also observed in an independent dataset (Supplementary Fig. 7B). Tumor myeloid cell compartments were particularly rich in monocyte-derived macrophage clusters 1 and 2. Of these, cluster 1 showed high expression of *SELENOP* (Supplementary Fig. 8B, arrowhead), which has been related to M2 polarization [20], and low scores of immune response-related signatures (Fig. 4C, white arrowheads), while cluster 2 expressed high levels of proinflammatory chemokines, such as *CXCL9* and *CXCL10*, the proinflammatory cytokine *IL1B* (Supplementary Fig. 8B, arrowheads), and gene signatures related to immune response and M1 polarization [21, 22] (Fig. 4C, black arrowheads). This indicates that monocyte-derived macrophage clusters 1 and 2 represent non- and proinflammatory functional states, respectively, which were inversely correlated across patients (Fig. 4D). In addition, the proportion of proinflammatory monocyte-derived macrophages cluster 2 correlated with other myeloid cell types such as plasmacytoid dendritic cells (Fig. 4D). The distribution of proinflammatory monocyte-derived macrophages cluster 2 and plasmacytoid dendritic cells was validated by immunostaining for CXCL9 and CD123, respectively (Fig. 4E, Supplementary Fig. 8D).

Within the tumor lymphoid cell compartment, CD8+ T, B, and plasma cells were increased, while NK and conventional T cells were decreased compared to normal tissue controls (Fig. 4A). Regulatory T cells were almost exclusively found in tumor tissue samples and expressed inhibitory molecules such as *CTLA4* and *TIGIT* (Supplementary Fig. 8C, arrowheads) corresponding to their immunosuppressive role [23]. We identified in total four clusters of CD8+ T cells (Fig. 4A). The tumor-enriched CD8+ T cell clusters 1 and 2 and proliferating CD8+ T cells were characterized by an exhaustion signature [10] (Fig. 4F, black arrowheads), while this score was low in the normal-enriched CD8+ T cell cluster 3 (Fig. 4F, white arrowhead). Exhausted CD8+ T cells were significantly associated with proinflammatory monocyte-derived macrophages (cluster 2) and plasmacytoid dendritic cell numbers (Fig. 4G), and a similar trend, albeit not significant, was seen for regulatory T cells.

Taken together, we identify patient-overarching changes of the immune cell composition from normal lung tissue to adenocarcinoma, and distinct tumor immune microenvironment patterns contributing to interpatient heterogeneity.

### The tumor microenvironment of lung adenocarcinoma features two major patterns

**Fig. 5:**
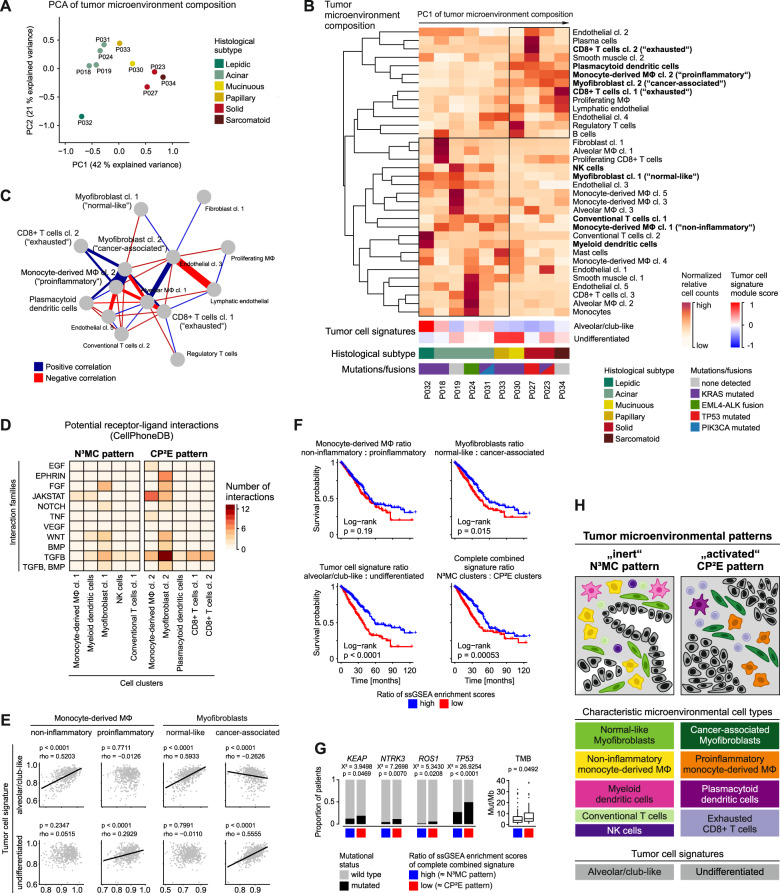
Tumor microenvironmental patterns in lung adenocarcinomas. **A** Principal component analysis based on the proportion of stromal and immune cell clusters, color-coded by histological subtype, patients indicated. **B** Normalized proportion of stromal and immune cell clusters, mean module scores of tumor cell signatures, histological subtypes and mutation status per patient, patients sorted along the first principal component from principal component analysis in (**A**), cell clusters included in the model in (**H**) in bold. **C** Correlation of the proportion of stromal and immune cell clusters, most connected section of correlation network plot shown; Spearman’s correlation statistics, only correlations with rho > 0.7 and *p* < 0.05 shown. **A**, **B**, **C** Cell clusters occurring in <3 patients were excluded from analyses. **D** Number of potential paracrine interactions from microenvironmental cell clusters to tumor cells of the N³MC or CP²E pattern, computed using CellPhoneDB, grouped by interaction families, color-coded by number of interactions (see also Supplementary Fig. 10). **E**–**G** Analysis of the TCGA lung adenocarcinoma cohort. **E** Correlation of ssGSEA enrichment scores based on marker genes of selected microenvironmental cell clusters and tumor cell signatures; *n* = 533, Spearman’s correlation statistics, linear regression line. **F** Kaplan–Meier overall survival curves, cases grouped by the ratio of ssGSEA enrichment scores of indicated microenvironmental cell clusters or tumor cell signatures or a combined signature encompassing all cell clusters of the N³MC or CP²E pattern, respectively; *n* = 524, log-rank statistics. **G** Proportion of patients with oncogenic mutations and tumor mutational burden (TMB), patients grouped by ratio of ssGSEA enrichment scores of the combined signature in (**F**); *n* = 525 for mutations, Chi-squared test, *n* = 242 for TMB, two-sided Welch’s *t* test. **H** Schematic representation of subtypes of lung adenocarcinoma characterized by different grades of tumor epithelial cell differentiation and different composition of the corresponding tumor microenvironment.

To integrate our analyses of variable cell prevalences in the tumor microenvironment, we calculated proportions of cells of the myeloid, lymphoid, endothelial and fibroblastic/muscle cell compartments across patients (for cell counts see Supplementary Tables 1–4). Principal component analysis showed that tumors formed subgroups based on the cellular composition of the tumor microenvironment (Fig. 5A). One group of tumors (P018, P019, P024, P031, P032, and P033) was marked by normal-like myofibroblasts, non-inflammatory monocyte-derived macrophages, NK cells, myeloid dendritic cells and conventional T cells, referred to as N³MC pattern (Fig. 5B, Supplementary Figs. 7C, 9A). A second group of tumors (P023, P027, P030 and P034) was characterized by cancer-associated myofibroblasts, proinflammatory monocyte-derived macrophages, plasmacytoid dendritic cells and exhausted CD8+ T cells, referred to as CP²E pattern (Fig. 5B, Supplementary Figs. 7C, 9A). Comparable patterns were also found in an independent single-cell dataset of primary lung adenocarcinoma (Supplementary Fig. 7D–F). The two microenvironmental patterns roughly separated tumors by histological grade (Fig. 5A, Supplementary Fig. 7C), by expression scores of “alveolar/club-like” versus “undifferentiated” tumor cell signatures (Fig. 5B), and by prevalence of microenvironmental cell clusters (Fig. 5B, C, Supplementary Fig. 9B).

We quantified potential receptor-ligand interactions within the N³MC and CP²E tumor microenvironments, which were most frequent between tumor cells and cancer-associated myofibroblast cluster 2 of the CP²E pattern (Supplementary Table 5). Focusing on the most relevant oncogenic pathways (Supplementary Table 6), we found that tumor cells in the CP²E environment receive potential paracrine signals from cancer-associated myofibroblast cluster 2 activating Ephrin, FGF, WNT, TGFβ, and BMP signaling, and from proinflammatory monocyte-derived macrophages cluster 2 potentially activating JAK/STAT signaling (Fig. 5D, Supplementary Fig. 10).

To test if tumor microenvironmental patterns can be recapitulated by bulk gene expression patterns in larger patient cohorts, we analyzed the expression of cell cluster marker genes in The Cancer Genome Atlas (TCGA) lung adenocarcinoma cohort encompassing 533 patients (for marker genes see Supplementary Tables 7–10). We found a specific positive correlation of the alveolar/club-like tumor cell signature with non-inflammatory monocyte-derived macrophages and normal-like myofibroblasts, recapitulating the N³MC pattern, and of the undifferentiated tumor cell signature with proinflammatory monocyte-derived macrophages and cancer-associated myofibroblasts, recapitulating the CP²E pattern (Fig. 5E, Supplementary Fig. 9C).

To investigate the biological and clinical relevance of these patient subgroups, we analyzed the overall survival of the TCGA lung adenocarcinoma cohort contigent on expression of the different gene signatures. N³MC-related gene signatures were associated with a better overall survival compared to signatures of the CP²E pattern (Fig. 5F). In multivariate analyses, this association was significant for the tumor cell signatures (HR 0.50, 95% CI 0.35-0.71, *p* = 0.0001). Although individual cell cluster signatures had different prognostic relevance, a combination of all cell cluster signatures as well as a simplified signature of 20 genes separated patients into prognostic subgroups (Fig. 5F, Supplementary Fig. 11A–C, Supplementary Table 11). The group with worse prognosis and higher CP²E scores was characterized by more frequent mutations in *KEAP*, *NTRK3*, *ROS1*, and *TP53* as well as higher mutational burden (Fig. 5G).

In summary, our results show that lung adenocarcinomas can be stratified into clinically relevant subgroups which are not only characterized by the grade of tumor epithelial cells, but also by the cellular composition of their associated tumor microenvironment (Fig. 5H). We therefore propose two major microenvironmental patterns in lung adenocarcinoma which we term N³MC and CP²E as akronyms of the respective characteristic cell types.

## Discussion

By applying single-cell RNA sequencing to lung adenocarcinomas, we identified two major microenvironmental patterns, referred to as N³MC and CP²E (Fig. 5H). We characterized the N³MC tumor microenvironment as rather inert and normal-like, as it was defined by normal-like myofibroblasts, conventional T cells, NK cells, non-inflammatory monocyte-derived macrophages and myeloid dendritic cells. The N³MC pattern was associated with an alveolar/club-like gene expression pattern of carcinoma cells, lower histological grade and better prognosis. In contrast, the CP²E tumor microenvironment was proinflammatory and characterized by proinflammatory monocyte-derived macrophages, plasmacytoid dendritic cells, exhausted CD8+ T cells and cancer-associated myofibroblasts. The CP²E pattern was associated with an undifferentiated gene expression pattern of carcinoma cells, higher histological grade, and worse prognosis.

In the clinic, tumor stage and histological grading are used to predict prognosis in lung adenocarcinoma. Grading is based on the predominant growth pattern but other histological features have been proposed in order to improve risk stratification [24, 25]. However, these concepts focus on the carcinoma cells and do not include features of the complex composition of the tumor microenvironment. Most available studies on prognostic microenvironmental features focus on single-cell types identified by immunostaining. Cancer-associated fibroblasts were found to correlate with worse prognosis using different biomarkers [26–28], while tumor-infiltrating T cells indicated better prognosis in multiple studies [29–31]. The prognostic value of proinflammatory tumor-associated macrophages (M1) is inconclusive across the available studies, likely because differentiation of macrophage subtypes by immunostaining is limited [32]. In order to account for the complexity of the tumor microenvironment, patterns based on tumor-infiltrating T cells and PD-L1 expression have been proposed [33, 34]. Beyond this, the cellular complexity has recently been studied by multiplexed immunostaining [35, 36]. Gene expression profiling of immune-related genes provide further functional insights, but have yet been limited to the use of bulk methods [37–39]. Single-cell RNA sequencing combines both a comprehensive census of microenvironmental cell types and an in-depth functional characterization of their transcriptional profiles. In our study, the microenvironmental CP²E pattern was associated with worse prognosis, which is in line with existing evidence for cancer-associated myofibroblasts [40] and plasmacytoid dendritic cells [41], but partly contradicts previous works with regard to proinflammatory tumor-associated macrophages [32]. The absence of cancer-associated myofibroblasts, and the presence of NK cells [42] and conventional T cells [31] support the association of the N³MC pattern with better prognosis.

Characterization of PD-L1 expression on tumor-infiltrating immune cells is already used to predict response to immune checkpoint inhibitors [43], but more comprehensive characterization of the microenvironment will likely provide even deeper insights for patient stratification and drug development. Recently, expression of the proinflammatory cytokines CXCL9 and CXCL10 by tumor-associated macrophages has been shown to be essential for tumor response to anti-PD-L1 therapy [44, 45]. High expression of these cytokines in proinflammatory monocyte-derived macrophages, high numbers of exhausted CD8+ T cells [46] and high mutational burden [47] indicates a predictive relevance of the CP²E microenvironment. In addition, the CP²E pattern was characterized by cancer-associated myofibroblasts expressing potential novel therapeutic targets, such as *PSTN* and *MMP11* [48, 49]. Despite an overall depletion of NK cells in tumors in agreement with other studies [7, 9], the N³MC microenvironmental pattern still contained small populations of NK cells which could potentially be targeted by immunostimulatory agents [50]. Furthermore, we confirmed enrichment of B cells in some tumors [8, 9] with potential importance in development of novel immunotherapies [51].

We envisage that single-cell approaches will also contribute to a better understanding of tumor cells with regard to their high mutational burden [52] and genomic intertumoral heterogeneity [53]. Squamous and adenocarcinomas of the lung can exhibit distinct transcriptional differences [54, 55], and interestingly, despite intertumoral heterogeneity, we found that tumor epithelial transcriptomes retained histology-related patterns that we termed “alveolar/club-like” and “undifferentiated” across patients which are reminiscent of recently defined signatures [9]. The “undifferentiated” tumor cell signature was characterized by high activity of TGFβ signaling and hypoxia-induced pathways, which are both known activators of epithelial-mesenchymal transition [56, 57], corresponding to poor histological differentiation of these tumors. The “alveolar/club-like” signature was characterized by PI3K signaling, an important oncogenic pathway promoting tumor growth and potentially affecting the tumor microenvironment by upregulating PD-L1 expression in carcinoma cells [58–60]. EGFR and JAK/STAT signaling, associated with the “undifferentiated” signature, and PI3K signaling, associated with the “alveolar/club-like” signature, are potential therapeutic targets [4, 60, 61]. We identified cancer-associated myofibroblasts as potential sources of TGFβ ligands in the CP²E pattern, while EGFR interactions were equally present in both patterns, underlining that signaling pathways in tumor cells can be activated extrinsically via paracrine signals from the microenvironment but also via other mechanisms, e.g., intrinsically via oncogenic mutations.

Single-cell approaches provide high-resolution information on the cellular complexity of tumor tissues but are limited in cohort size. We validated some findings from single-cell RNA sequencing by immunostainings and identified a prognostic set of 20 genes recapitulating microenvironmental patterns. Alternative methods, such as multiplex immunostaining or bulk gene expression profiling, will facilitate validation of findings in large cohorts and translation into clinical application. So far, our findings suggest a prognostic relevance of tumor microenvironmental patterns which could aid in therapeutical decision making. Moreover, preclinical findings from other groups suggest that tumor microenvironmental patterns could be associated with differential responses to immune checkpoint inhibition, thus distinct microenvironmental features such as tumor-infiltrating CD8+ T cells or CXCL9+ macrophages could complement current patient stratification by PD-L1 expression. Finally, characterization of the tumor microenvironment on single-cell resolution can provide insight on possible novel therapeutic targets. It remains an open question to which extent cancer cells shape their microenvironment and to which extent the microenvironment affects cancer cells. Preclinical efforts need to be complemented by translational studies to identify critical mechanisms in this complex network that determine tumor response to targeted or immune therapies in the clinical context. While our study demonstrates how single-cell gene expression profiling of clinical samples can contribute to this task, in the future, other single-cell approaches comprising spatial information [62], surface protein expression [63], and epigenetic characterization [64] will help to complete the picture [65].

## Methods

### Collection of tissue specimens

Fresh normal lung parenchyma and tumor tissues were obtained during intraoperative pathologist consultation from previously untreated lung adenocarcinoma patients undergoing primary surgery. Patients were aware of the planned research and agreed to the use of tissue. Research was approved by vote EA4/164/19 of the ethics committee of Charité—Universitätsmedizin Berlin.

### Tissue dissociation and single-cell isolation

Tissue specimens of ~0.1–0.5 cm³ were stored on ice in Tissue Storage Solution (Miltenyi, Bergisch Gladbach, Germany) for transport. Tissues were minced, dissociated using the Tumor Dissociation Kit, human (Miltenyi) and a gentleMACS Octo Dissociator with heaters (Miltenyi), using program 37C_h_TDK_1 for 30–45 min. Subsequent steps were performed at 4 °C. Cell suspensions were filtered using 100 µm filters, pelleted by centrifugation at 300 × *g* in BSA-coated low-binding tubes, treated with 1 ml ACK erythrocyte lysis buffer for 1 min, washed with DMEM, pelleted, resuspended in PBS, filtered using 20 µm filters, debris was removed using the Debris Removal Solution (Miltenyi), and cells were counted using a Neubauer chamber.

### Single-cell RNA sequencing

10,000 single cells were used for single-cell library production, using the Chromium Single Cell 3′ Reagent Kit v3 and the Chromium Controller (10x Genomics, Pleasanton, California, USA) according to the manufacturer’s protocol. Libraries were sequenced on a HiSeq 4000 Sequencer (Illumina, San Diego, California, USA) at in average 54,000 reads per cell.

### H&E and immunostaining

3–5 µm tissue sections of formalin-fixed and paraffin-embedded (FFPE) tissue were prepared. For H&E staining, sections were stained for 8 min in acidic haemalum staining solution (Waldeck, Münster, Germany) and for 2.5 min in eosin staining solution (Sigma-Aldrich, St. Louis, Missouri, USA) using a Tissue-Tek Prisma Plus slide stainer (Sakura, Staufen im Breisgau, Germany).

Immunohistochemical stainings were performed on the BenchMark XT immunostainer (Ventana, Oro Valley, Arizona, USA). Tissue sections were incubated in CC1 or CC2 buffer (Ventana) for 30 min at 100 °C, and subsequently with primary antibodies for 60 min and with secondary antibodies for 30 min at room temperature. Multiplex immunofluorescence stainings were performed using t-CyCIF as described in [66]. Primary antibodies are listed in Supplementary Table 12. H&E and immunohistochemical images were taken using a Pannoramic SCAN 150 scanner (3DHISTECH, Budapest, Hungary), immunofluorescence images were taken using a CQ1 microscope (Yokogawa, Musashino, Japan). For quantification, 10 random areas of 0.48 mm² each were analyzed by a pathologist. CD123- and CXCL9-positve cells were counted per area. For CTHRC1 quantification, using the ImageJ software, stromal area was manually marked as region of interest, color channels were split by “Color deconvolution”, the DAB channel was binarized using an intensity threshold of 150, and the proportion of area covered by positive signal was measured per image.

### Panel sequencing

For panel sequencing, tumor-enriched areas were macrodissected from FFPE tissue sections.

DNA was isolated using the Maxwell RSC DNA FFPE Kit (Promega, Madison, Wisconsin, USA) on a Maxwell RSC 48 Instrument (Promega) and analyzed using the nNGM panel v1 (ThermoFisher, Waltham, Massachusetts, USA), an Ion 530 chip (ThermoFisher) and the Ion Chef/Ion S5 XL System (ThermoFisher).

RNA was isolated using the Maxwell RSC RNA FFPE Kit (Promega) on a Maxwell RSC 48 Instrument (Promega) and analyzed using the Oncomine Focus RNA Assay (ThermoFisher), an Ion 530 chip and the Ion Chef/Ion S5 XL System (ThermoFisher).

The Sequence Pilot Software (Version 4.4.0, JSI Medical Systems) and the Ion Reporter Software (Version 5.12, ThermoFisher) were used for variant calling.

### Single-cell RNA sequencing data analysis

#### Preprocessing, filtering, and normalization

UMIs were quantified using Cellranger 3.0.2 (10x Genomics) with reference transcriptome GRCh38. Subsequent analyses were performed using “Seurat v3” [67], if not stated otherwise. Single-cell gene expression data of all patients were merged, and transcriptomes were filtered for cells with 500–10,000 genes detected, 1000–100,000 UMIs counted, fraction of mitochondrial reads <30%, and fraction of hemoglobin reads <5%. After filtering, UMI counts were variance-stabilized using scTransform with 3000 variable features [68], while regressing out number of UMIs and fraction of mitochondrial reads.

#### Clustering and cell type annotation

Top 15 principal components were used to construct SNN graph and UMAP embedding. Main cell types were identified by scoring canonical cell type markers across clusters. PCA, SNN graph construction and UMAP embedding was rerun on main cell type subsets. Cell type markers were adapted from Habermann et al. [69]. and Tata and Rajagopal [70] (Supplementary Table 13). Cell type signatures from Vieira Braga et al. [13] and Travaglini et al. [14] were used to validate manual cell type annotation. Epithelial or immune contaminated clusters were identified by *EPCAM* or *PTPRC*, respectively, and removed before further analyses. DoubletFinder v2.0 was used to estimate cell doublet distribution (Supplementary Fig. 1D). Epithelial cell clusters overrepresented in tumor tissue samples were annotated as tumor cells. For copy-number assignment, InferCNV v1.3.3 was used with default parameters.

#### Differential gene expression analysis

Marker genes for each cell cluster versus all cells of the respective subset were computed using the FindAllMarkers function with the following parameters: only positive markers, fraction of expressing cells inside the cluster ≥0.25, difference between fraction of expressing cells inside and outside the cluster ≥0.25, log fold change between cells inside and outside the cluster ≥0.25.

#### Functional analysis

Cell cycle phases were scored as implemented in “Seurat v3”. Expression of gene sets of the Hallmark signature collection of the Broad Institute [21], and M1 vs. M2 up- and downregulated genes [22] were scored using the AddModuleScore function. Oncogenic signaling pathway activity scores were computed using the R package “progeny” [71, 72]. Potential paracrine interactions were computed using the “CellPhoneDB” toolkit with default parameters [73].

### Analysis of the TCGA lung adenocarcinoma cohort

FPKM-normalized gene expression values of 533 lung adenocarcinoma (LUAD) cases were downloaded using the R package “TCGAbiolinks” [74] and log2 transformed. Marker genes from myeloid, lymphoid, endothelial, stromal cell subsets, and the “alveolar/club-like” and “undifferentiated” tumor cell signatures were used as gene sets to perform single-sample gene set enrichment analysis (ssGSEA) [75] on TCGA LUAD gene expression data using the R package “GSVA” [76]. Associations between enrichment scores (ES) from ssGSEA were calculated using the R package “corrplot”.

Next, patients were grouped by dichotomization of ssGSEA ES (> or ≤median). Survival data of the TCGA LUAD cohort was available for 524 cases and downloaded using the R package “TCGAbiolinks” [74]. Survival curves, log-rank statistics and Cox regression were calculated using the R packages “survival” and “survminer”. Data on mutations and mutational burden was available for 525 and 242 cases, respectively.

### Statistical analysis

Patient groups were compared using the Welch’s *t* test or Mann–Whitney *U* test (both two-sided, unequal variances), as indicated, after testing for normal distribution using the Shapiro–Wilk’s test. Distribution of mutations across patient groups was analyzed using the Chi-squared test. For correlation analysis, we calculated the Pearson or Spearman correlation coefficient, as indicated. *P* values < 0.05 were considered significant.

## Supplementary information

Supplementary Figures

Supplementary Table 1

Supplementary Table 2

Supplementary Table 3

Supplementary Table 4

Supplementary Table 5

Supplementary Table 6

Supplementary Table 7

Supplementary Table 8

Supplementary Table 9

Supplementary Table 10

Supplementary Table 11

Supplementary Table 12

Supplementary Table 13

## Data Availability

Processed count data used for analyses is available as a Code Ocean capsule from 10.24433/CO.0121060.v1.

## Code availability

Code used for analyses is available as a Code Ocean capsule from 10.24433/CO.0121060.v1. Complementary code is available from https://github.com/bischofp/single_cell_lung_adenocarcinoma.

## References

[1] Bray F, Ferlay J, Soerjomataram I, Siegel RL, Torre LA, Jemal A (2018). Global cancer statistics 2018: GLOBOCAN estimates of incidence and mortality worldwide for 36 cancers in 185 countries. CA Cancer J Clin.

[2] Wakelee HA, Chang ET, Gomez SL, Keegan TH, Feskanich D, Clarke CA (2007). Lung cancer incidence in never smokers. J Clin Oncol.

[3] Cancer Genome Atlas Research N. (2014). Comprehensive molecular profiling of lung adenocarcinoma. Nature..

[4] Hirsch FR, Scagliotti GV, Mulshine JL, Kwon R, Curran WJ, Wu YL (2017). Lung cancer: current therapies and new targeted treatments. Lancet..

[5] Memon H, Patel BM (2019). Immune checkpoint inhibitors in non-small cell lung cancer: a bird’s eye view. Life Sci.

[6] Altorki NK, Markowitz GJ, Gao D, Port JL, Saxena A, Stiles B (2019). The lung microenvironment: an important regulator of tumour growth and metastasis. Nat Rev Cancer.

[7] Lavin Y, Kobayashi S, Leader A, Amir ED, Elefant N, Bigenwald C (2017). Innate immune landscape in early lung adenocarcinoma by paired single-cell analyses. Cell..

[8] Lambrechts D, Wauters E, Boeckx B, Aibar S, Nittner D, Burton O (2018). Phenotype molding of stromal cells in the lung tumor microenvironment. Nat Med.

[9] Kim N, Kim HK, Lee K, Hong Y, Cho JH, Choi JW (2020). Single-cell RNA sequencing demonstrates the molecular and cellular reprogramming of metastatic lung adenocarcinoma. Nat Commun.

[10] Guo X, Zhang Y, Zheng L, Zheng C, Song J, Zhang Q (2018). Global characterization of T cells in non-small-cell lung cancer by single-cell sequencing. Nat Med.

[11] Becht E, McInnes L, Healy J, Dutertre CA, Kwok IWH, Ng LG, (2019). Dimensionality reduction for visualizing single-cell data using UMAP. Nat Biotechnol..

[12] Lee HO, Hong Y, Etlioglu HE, Cho YB, Pomella V, Van den Bosch B, (2020). Lineage-dependent gene expression programs influence the immune landscape of colorectal cancer. Nat Genet.

[13] Vieira Braga FA, Kar G, Berg M, Carpaij OA, Polanski K, Simon LM (2019). A cellular census of human lungs identifies novel cell states in health and in asthma. Nat Med.

[14] Travaglini KJ, Nabhan AN, Penland L, Sinha R, Gillich A, Sit RV (2020). A molecular cell atlas of the human lung from single-cell RNA sequencing. Nature.

[15] Naizhen X, Kido T, Yokoyama S, Linnoila RI, Kimura S (2019). Spatiotemporal expression of three secretoglobin proteins, SCGB1A1, SCGB3A1, and SCGB3A2, in mouse airway epithelia. J Histochem Cytochem.

[16] Yoshida GJ (2020). Regulation of heterogeneous cancer-associated fibroblasts: the molecular pathology of activated signaling pathways. J Exp Clin Cancer Res.

[17] Petrova V, Annicchiarico-Petruzzelli M, Melino G, Amelio I (2018). The hypoxic tumour microenvironment. Oncogenesis..

[18] Franklin RA, Li MO (2016). Ontogeny of tumor-associated macrophages and its implication in cancer regulation. Trends Cancer.

[19] Solberg R, Smith R, Almlof M, Tewolde E, Nilsen H, Johansen HT (2015). Legumain expression, activity and secretion are increased during monocyte-to-macrophage differentiation and inhibited by atorvastatin. Biol Chem.

[20] Solinas G, Schiarea S, Liguori M, Fabbri M, Pesce S, Zammataro L (2010). Tumor-conditioned macrophages secrete migration-stimulating factor: a new marker for M2-polarization, influencing tumor cell motility. J Immunol.

[21] Liberzon A, Birger C, Thorvaldsdottir H, Ghandi M, Mesirov JP, Tamayo P (2015). The Molecular Signatures Database (MSigDB) hallmark gene set collection. Cell Syst..

[22] Martinez FO, Gordon S, Locati M, Mantovani A (2006). Transcriptional profiling of the human monocyte-to-macrophage differentiation and polarization: new molecules and patterns of gene expression. J Immunol.

[23] Jiang Y, Li Y, Zhu B (2015). T-cell exhaustion in the tumor microenvironment. Cell Death Dis.

[24] Travis WD, Brambilla E, Nicholson AG, Yatabe Y, Austin JHM, Beasley MB (2015). The 2015 World Health Organization classification of lung tumors: impact of genetic, clinical and radiologic advances since the 2004 classification. J Thorac Oncol.

[25] Moreira AL, Ocampo PSS, Xia Y, Zhong H, Russell PA, Minami Y (2020). A grading system for invasive pulmonary adenocarcinoma: a proposal from the International Association for the Study of Lung Cancer Pathology Committee. J Thorac Oncol.

[26] Kubouchi Y, Yurugi Y, Wakahara M, Sakabe T, Haruki T, Nosaka K (2018). Podoplanin expression in cancer-associated fibroblasts predicts unfavourable prognosis in patients with pathological stage IA lung adenocarcinoma. Histopathology..

[27] Shi J, Hou Z, Yan J, Qiu W, Liang L, Meng M (2020). The prognostic significance of fibroblast activation protein-alpha in human lung adenocarcinoma. Ann Transl Med.

[28] Inoue C, Tamatsuki D, Miki Y, Saito R, Okada Y, Sasano H (2019). Prognostic significance of combining immunohistochemical markers for cancer-associated fibroblasts in lung adenocarcinoma tissue. Virchows Arch.

[29] Schalper KA, Brown J, Carvajal-Hausdorf D, McLaughlin J, Velcheti V, Syrigos KN, (2015). Objective measurement and clinical significance of TILs in non-small cell lung cancer. J Natl Cancer Inst.

[30] Donnem T, Hald SM, Paulsen EE, Richardsen E, Al-Saad S, Kilvaer TK (2015). Stromal CD8+ T-cell density—a promising supplement to TNM staging in non-small cell lung cancer. Clin Cancer Res.

[31] Geng Y, Shao Y, He W, Hu W, Xu Y, Chen J (2015). Prognostic role of tumor-infiltrating lymphocytes in lung cancer: a meta-analysis. Cell Physiol Biochem.

[32] Conway EM, Pikor LA, Kung SH, Hamilton MJ, Lam S, Lam WL (2016). Macrophages, inflammation, and lung cancer. Am J Respir Crit Care Med.

[33] Teng MW, Ngiow SF, Ribas A, Smyth MJ (2015). Classifying cancers based on T-cell Infiltration and PD-L1. Cancer Res.

[34] Lin Z, Gu J, Cui X, Huang L, Li S, Feng J (2019). Deciphering microenvironment of NSCLC based on CD8+ TIL density and PD-1/PD-L1 expression. J Cancer.

[35] Parra ER, Villalobos P, Behrens C, Jiang M, Pataer A, Swisher SG (2018). Effect of neoadjuvant chemotherapy on the immune microenvironment in non-small cell lung carcinomas as determined by multiplex immunofluorescence and image analysis approaches. J Immunother Cancer.

[36] Peng H, Wu X, Zhong R, Yu T, Cai X, Liu J, et al. Profiling tumor immune microenvironment of non-small cell lung cancer using multiplex immunofluorescence. bioRxiv. 2021. 10.1101/2021.05.28.446005. 10.3389/fimmu.2021.750046 PMC8600321 34804034

[37] Tao Y, Li Y, Liang B (2020). Comprehensive analysis of microenvironment-related genes in lung adenocarcinoma. Future Oncol.

[38] Ojlert AK, Halvorsen AR, Nebdal D, Lund-Iversen M, Solberg S, Brustugun OT (2019). The immune microenvironment in non-small cell lung cancer is predictive of prognosis after surgery. Mol Oncol.

[39] Huang J, Li J, Zheng S, Lu Z, Che Y, Mao S (2020). Tumor microenvironment characterization identifies two lung adenocarcinoma subtypes with specific immune and metabolic state. Cancer Sci.

[40] Ito M, Ishii G, Nagai K, Maeda R, Nakano Y, Ochiai A (2012). Prognostic impact of cancer-associated stromal cells in patients with stage I lung adenocarcinoma. Chest..

[41] Koucky V, Boucek J, Fialova A (2019). Immunology of plasmacytoid dendritic cells in solid tumors: a brief review. Cancers.

[42] Zhang S, Liu W, Hu B, Wang P, Lv X, Chen S (2020). Prognostic significance of tumor-infiltrating natural killer cells in solid tumors: a systematic review and meta-analysis. Front Immunol.

[43] Herbst RS, Giaccone G, de Marinis F, Reinmuth N, Vergnenegre A, Barrios CH (2020). Atezolizumab for first-line treatment of PD-L1-selected patients with NSCLC. N Engl J Med.

[44] Qu Y, Wen J, Thomas G, Yang W, Prior W, He W (2020). Baseline frequency of inflammatory Cxcl9-expressing tumor-associated macrophages predicts response to avelumab treatment. Cell Rep.

[45] House IG, Savas P, Lai J, Chen AXY, Oliver AJ, Teo ZL (2020). Macrophage-derived CXCL9 and CXCL10 are required for antitumor immune responses following immune checkpoint blockade. Clin Cancer Res.

[46] Jiang W, He Y, He W, Wu G, Zhou X, Sheng Q (2020). Exhausted CD8+T cells in the tumor immune microenvironment: new pathways to therapy. Front Immunol.

[47] Sholl LM, Hirsch FR, Hwang D, Botling J, Lopez-Rios F, Bubendorf L (2020). The promises and challenges of tumor mutation burden as an immunotherapy biomarker: a perspective from the International Association for the Study of Lung Cancer Pathology Committee. J Thorac Oncol.

[48] Okazaki T, Tamai K, Shibuya R, Nakamura M, Mochizuki M, Yamaguchi K (2018). Periostin is a negative prognostic factor and promotes cancer cell proliferation in non-small cell lung cancer. Oncotarget..

[49] Yang H, Jiang P, Liu D, Wang HQ, Deng Q, Niu X (2019). Matrix metalloproteinase 11 is a potential therapeutic target in lung adenocarcinoma. Mol Ther Oncolytics.

[50] Wrangle JM, Velcheti V, Patel MR, Garrett-Mayer E, Hill EG, Ravenel JG (2018). ALT-803, an IL-15 superagonist, in combination with nivolumab in patients with metastatic non-small cell lung cancer: a non-randomised, open-label, phase 1b trial. Lancet Oncol.

[51] Patel AJ, Richter A, Drayson MT, Middleton GW (2020). The role of B lymphocytes in the immuno-biology of non-small-cell lung cancer. Cancer Immunol Immunother.

[52] Alexandrov LB, Nik-Zainal S, Wedge DC, Aparicio SA, Behjati S, Biankin AV (2013). Signatures of mutational processes in human cancer. Nature..

[53] Liu Y, Zhang J, Li L, Yin G, Zhang J, Zheng S (2016). Genomic heterogeneity of multiple synchronous lung cancer. Nat Commun.

[54] Niemira M, Collin F, Szalkowska A, Bielska A, Chwialkowska K, Reszec J (2019). Molecular signature of subtypes of non-small-cell lung cancer by large-scale transcriptional profiling: identification of key modules and genes by weighted gene co-expression network analysis (WGCNA). Cancers.

[55] Molina-Romero C, Rangel-Escareno C, Ortega-Gomez A, Alanis-Funes GJ, Aviles-Salas A, Avila-Moreno F (2017). Differential gene expression profiles according to the Association for the Study of Lung Cancer/American Thoracic Society/European Respiratory Society histopathological classification in lung adenocarcinoma subtypes. Hum Pathol.

[56] Hao Y, Baker D, Ten Dijke P (2019). TGF-beta-mediated epithelial-mesenchymal transition and cancer metastasis. Int J Mol Sci.

[57] Tam SY, Wu VWC, Law HKW (2020). Hypoxia-induced epithelial-mesenchymal transition in cancers: HIF-1alpha and beyond. Front Oncol.

[58] Lastwika KJ, Wilson W, Li QK, Norris J, Xu H, Ghazarian SR (2016). Control of PD-L1 expression by oncogenic activation of the AKT-mTOR pathway in non-small cell lung cancer. Cancer Res.

[59] Gao Y, Yang J, Cai Y, Fu S, Zhang N, Fu X (2018). IFN-gamma-mediated inhibition of lung cancer correlates with PD-L1 expression and is regulated by PI3K-AKT signaling. Int J Cancer.

[60] Tan AC (2020). Targeting the PI3K/Akt/mTOR pathway in non-small cell lung cancer (NSCLC). Thorac Cancer.

[61] Mohrherr J, Haber M, Breitenecker K, Aigner P, Moritsch S, Voronin V (2019). JAK-STAT inhibition impairs K-RAS-driven lung adenocarcinoma progression. Int J Cancer.

[62] Stahl PL, Salmen F, Vickovic S, Lundmark A, Navarro JF, Magnusson J (2016). Visualization and analysis of gene expression in tissue sections by spatial transcriptomics. Science..

[63] Stoeckius M, Hafemeister C, Stephenson W, Houck-Loomis B, Chattopadhyay PK, Swerdlow H (2017). Simultaneous epitope and transcriptome measurement in single cells. Nat Methods.

[64] Buenrostro JD, Wu B, Litzenburger UM, Ruff D, Gonzales ML, Snyder MP (2015). Single-cell chromatin accessibility reveals principles of regulatory variation. Nature..

[65] Rajewsky N, Almouzni G, Gorski SA, Aerts S, Amit I, Bertero MG (2020). LifeTime and improving European healthcare through cell-based interceptive medicine. Nature.

[66] Lin JR, Izar B, Wang S, Yapp C, Mei S, Shah PM (2018). Highly multiplexed immunofluorescence imaging of human tissues and tumors using t-CyCIF and conventional optical microscopes. Elife.

[67] Stuart T, Butler A, Hoffman P, Hafemeister C, Papalexi E, Mauck WM (2019). Comprehensive integration of single-cell data. Cell..

[68] Hafemeister C, Satija R (2019). Normalization and variance stabilization of single-cell RNA-seq data using regularized negative binomial regression. Genome Biol.

[69] Habermann AC, Gutierrez AJ, Bui LT, Yahn SL, Winters NI, Calvi CL (2020). Single-cell RNA sequencing reveals profibrotic roles of distinct epithelial and mesenchymal lineages in pulmonary fibrosis. Sci Adv.

[70] Tata PR, Rajagopal J (2017). Plasticity in the lung: making and breaking cell identity. Development..

[71] Schubert M, Klinger B, Klunemann M, Sieber A, Uhlitz F, Sauer S (2018). Perturbation-response genes reveal signaling footprints in cancer gene expression. Nat Commun.

[72] Holland CH, Tanevski J, Perales-Paton J, Gleixner J, Kumar MP, Mereu E (2020). Robustness and applicability of transcription factor and pathway analysis tools on single-cell RNA-seq data. Genome Biol.

[73] Efremova M, Vento-Tormo M, Teichmann SA, Vento-Tormo R (2020). CellPhoneDB: inferring cell-cell communication from combined expression of multi-subunit ligand-receptor complexes. Nat Protoc.

[74] Colaprico A, Silva TC, Olsen C, Garofano L, Cava C, Garolini D (2016). TCGAbiolinks: an R/Bioconductor package for integrative analysis of TCGA data. Nucleic Acids Res.

[75] Barbie DA, Tamayo P, Boehm JS, Kim SY, Moody SE, Dunn IF (2009). Systematic RNA interference reveals that oncogenic KRAS-driven cancers require TBK1. Nature..

[76] Hanzelmann S, Castelo R, Guinney J (2013). GSVA: gene set variation analysis for microarray and RNA-seq data. BMC Bioinform.

