# Supplementary material for: Single-cell RNA sequencing reveals distinct tumor microenvironmental patterns in lung adenocarcinoma
Source: Oncogene. 2021 Oct 18;40(50):6748–58. doi: 10.1038/s41388-021-02054-3 (PMC8677623; doi:10.1038/s41388-021-02054-3)
Supplement: Supplementary file 1 — Supplementary Figures [file 41388_2021_2054_MOESM1_ESM.pdf]

Supplemental Figures

Single-cell RNA sequencing reveals distinct tumor microenvironmental patterns in lung adenocarcinoma

Philip Bischoff[1,2], Alexandra Trinks[1,2], Benedikt Obermayer[3], Jan Patrick Pett[3], Jennifer Wiederspahn[1,4], Florian Uhlitz[4,5], Xizi Liang[1], Annika Lehmann[1], Philipp Jurmeister[1,2,5], Aron Elsner[6], Tomasz Dziodzio[2,6], Jens-Carsten Rückert[6], Jens Neudecker[6], Christine Falk[7,8], Dieter Beule[3], Christine Sers[1,2,5], Markus Morkel[1,2,5], David Horst[1,2,5], Nils Blüthgen[1,2,4,5,9], Frederick Klauschen[1,5,9]

Affiliations:

1) Charité - Universitätsmedizin Berlin, corporate member of Freie Universität Berlin and Humboldt-Universität zu Berlin, Institute of Pathology, Charitéplatz 1, 10117 Berlin, Germany
2) Berlin Institute of Health at Charité - Universitätsmedizin Berlin, Charitéplatz 1, 10117 Berlin, Germany
3) Berlin Institute of Health at Charité - Universitätsmedizin Berlin, Core Unit Bioinformatics, Charitéplatz 1, 10117 Berlin, Germany
4) IRI Life Sciences, Humboldt University of Berlin, Philippstrasse 13, 10115 Berlin, Germany
5) German Cancer Consortium (DKTK), Partner Site Berlin, and German Cancer Research Center (DKFZ), 69120 Heidelberg, Germany
6) Charité - Universitätsmedizin Berlin, corporate member of Freie Universität Berlin and Humboldt-Universität zu Berlin, Department of Surgery, Campus Charité Mitte and Campus Virchow-Klinikum, Charitéplatz 1, 10117 Berlin, Germany
7) Institute of Transplant Immunology, Hannover Medical School, Carl-Neuberg-Str. 1, 30625 Hannover, Germany
8) DZIF, German Center for Infectious Diseases, TTU-IICH Hannover-Braunschweig site, 38124 Braunschweig, Germany
9) joint last authors

Corresponding author: Philip Bischoff, philip.bischoff@charite.de, +49 30 450 536 127

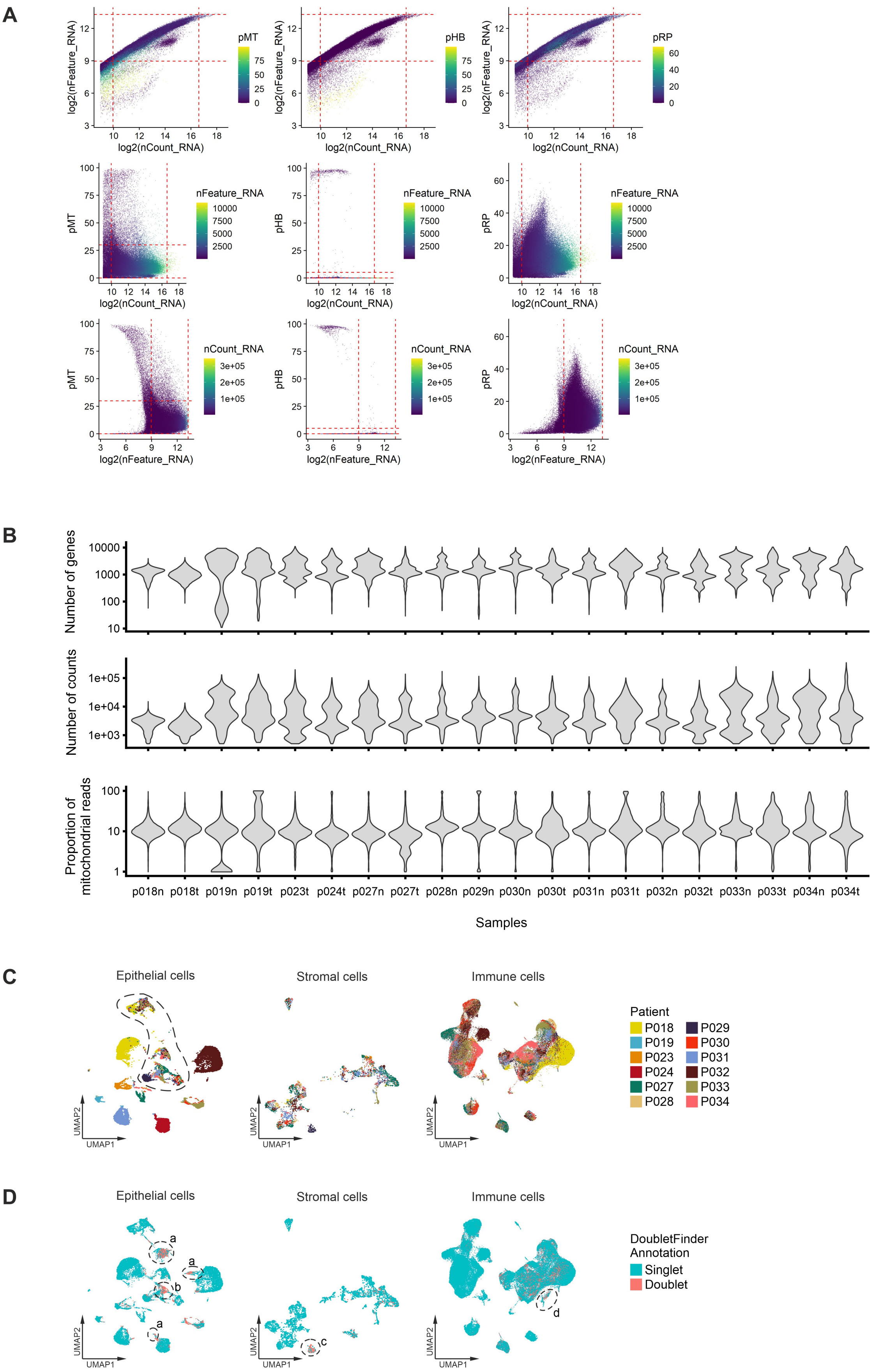

A
B
Number of genes
Number of counts
Proportion of mitochondrial reads
p018n p018t p019n p019t p023t p024t p027n p027t p028n p029n p030n p030t p031n p031t p032n p032t p033n p033t p034n p034t
Samples
C
Epithelial cells
Stromal cells
Immune cells
Patient
P018 P019 P023 P024 P027 P028 P029 P030 P031 P032 P033 P034
D
DoubletFinder Annotation
Singlet
Doublet
UMAP1
UMAP2

Supplemental Figure 1

(A) Scatter plots of quality control parameters of all single-cell transcriptomes analyzed in the study, red dashed lines indicate cut-off values for filtering of high-quality transcriptomes. (B) Violin plots of selected quality control parameters grouped by tissue sample, n = normal tissue, t = tumor tissue. (C) UMAPs of epithelial, stromal and immune single-cell transcriptomes, color-coded by patients. In epithelial transcriptomes, clusters predominantly originating from normal tissues are encircled in black. (D) UMAPs of epithelial, stromal and immune single-cell transcriptomes, color-coded by cell doublet assignment using DoubletFinder v2. Clusters ‘a’ have been removed because of coexpression of the immune marker gene *PTPRC*, cluster ‘b’ represents normal epithelial cells, cluster ‘c’ represents normal endothelial cells, cluster ‘d’ has been removed because of coexpression of the epithelial marker gene *EPCAM*. Taken together, increased numbers of cell doublets were only found in cell clusters, which are not relevant for further analyses of microenvironmental patterns.

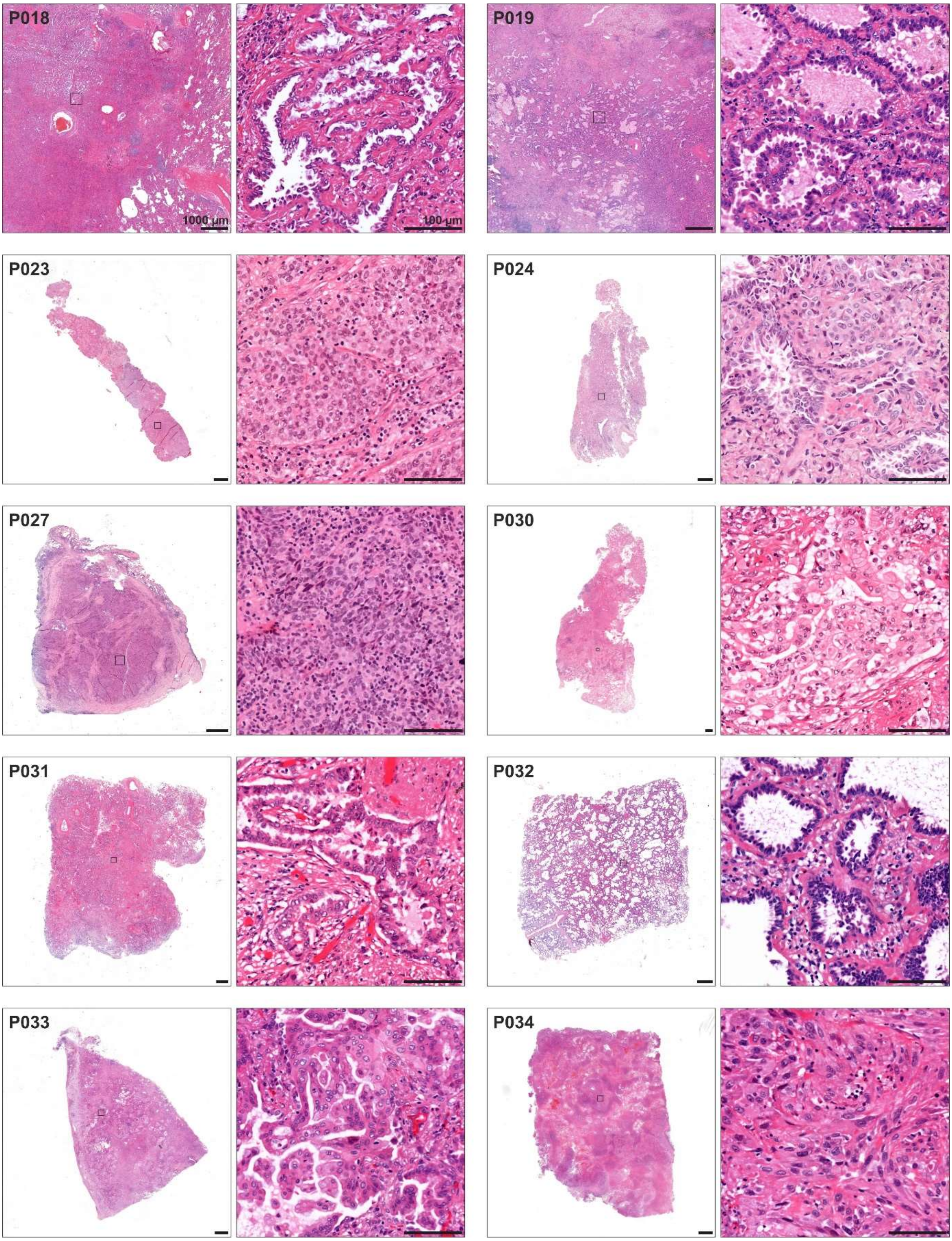

Supplemental Figure 2

H&E staining of lung adenocarcinomas analyzed in the study. For patients P018 and P019, representative tumor tissue shown, for all other patients, consecutive tissue sections of the sample used to prepare single-cell suspensions is shown. Black squares indicate magnified areas. Scale bars indicate 1000 µm or 100 µm in overview or magnified images, respectively.

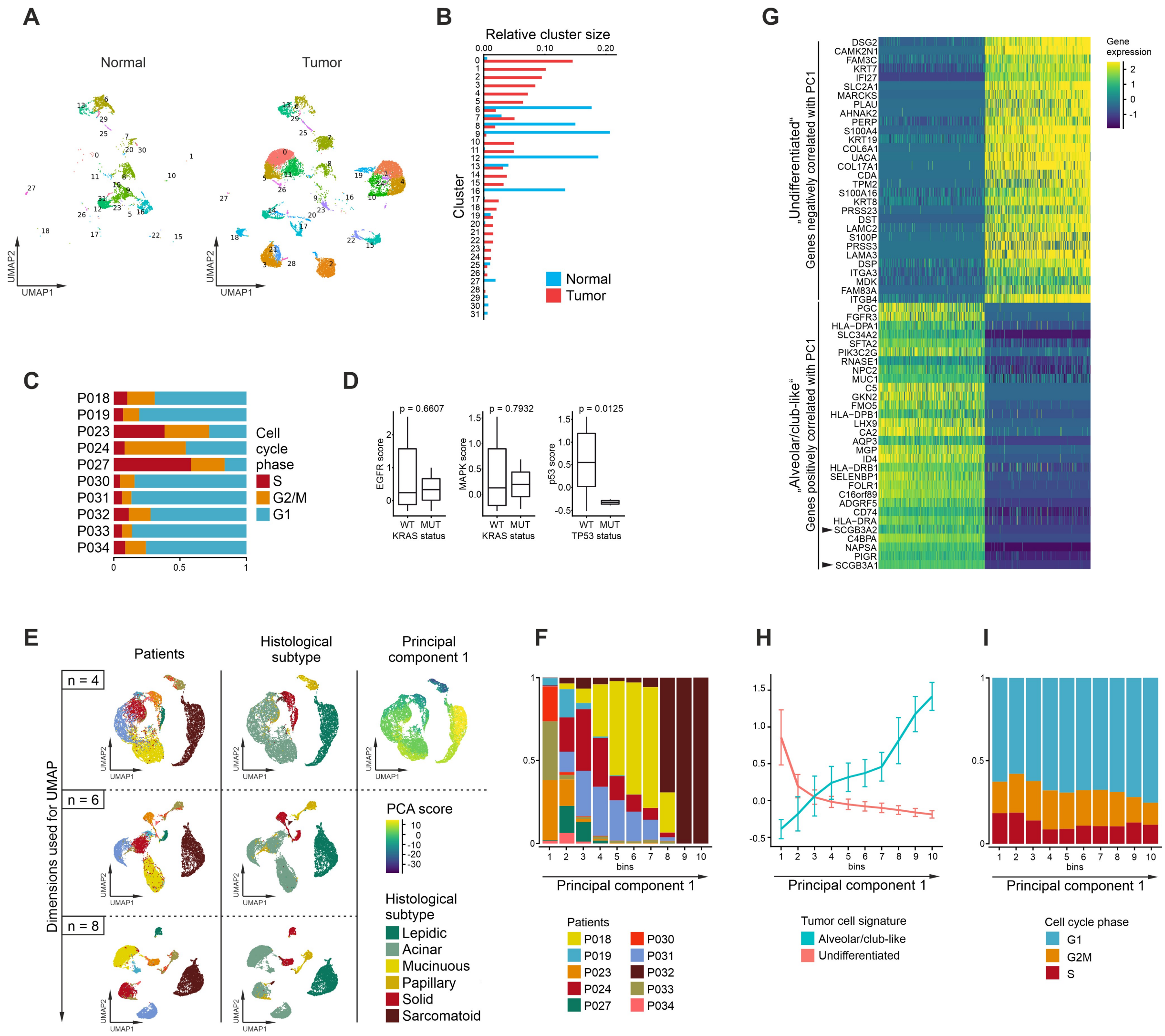

Supplemental Figure 3

(A) UMAPs based on the top 20 principal components of all epithelial single-cell transcriptomes split by tissue type, color-coded by cluster, cluster number indicated. (B) Relative size of clusters within normal (blue) or tumor (red) tissue samples. Clusters overrepresented in normal or tumor tissue samples were defined as normal or tumor cell clusters, repectively. (C) Quantification of cell cycle phase of tumor epithelial cells, grouped by patients. (D) Pathway activity scores of selected signaling pathways in *KRAS* wildtype and mutated, or *TP53* wildtype and mutated patients, respectively; n = 10, two-sided Welch's t-test statistics. (E) UMAPs of tumor epithelial cells based on the top 4, 6, or 8 principal components, color-coded by patient, histological subtype, or the score of principal component 1; for patient color code see (F). (F) Distribution of patient origin of tumor epithelial cells sorted along principal component 1. (G) Heatmap of the top 30 genes positively or negatively correlated with principal component 1 which were defined as the "alveolar/club-like" and "undifferentiated" gene signature, respectively, shown for the top 500 cells with the highest or lowest PCA scores, respectively. (H) Mean module scores of the tumor cell signatures based on the top 30 genes positively or negatively correlated with principal component 1 (see (G)), respectively, in tumor epithelial cells sorted along principal component 1, mean per bin ± s.d. (I) Distribution of cell cycle phase of tumor epithelial cells sorted along principal component 1.

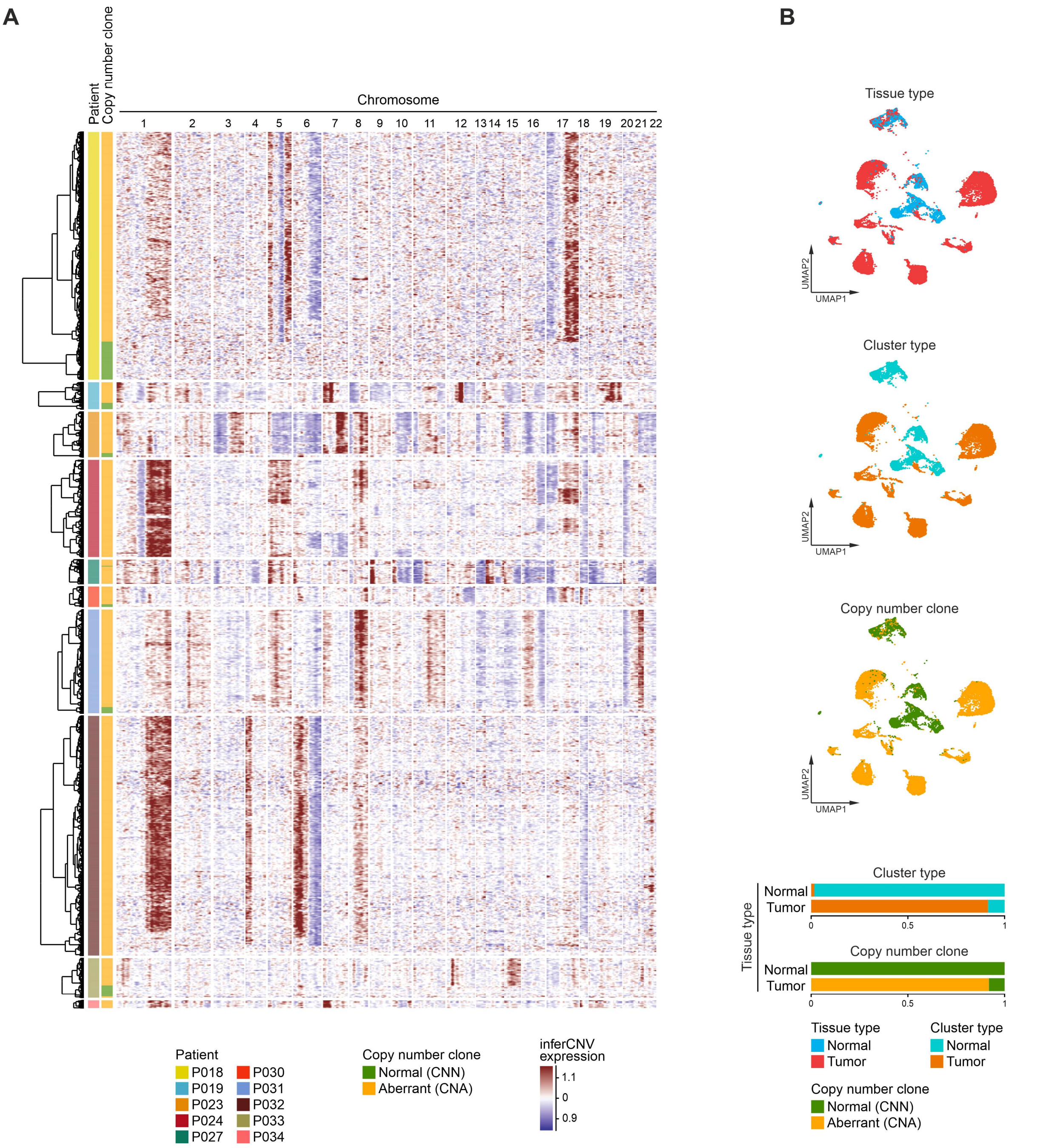

Supplemental Figure 4

(A) Copy-number aberrations in tumor tissue samples inferred from single-cell transcriptomes using inferCNV. Copy number aberrant clones were cut at k = 4 in inferCNV dendrograms. Clone-wise SCNA scores were computed by calculating the ratio of average standard deviation in inferCNV expression of all cells and all normal samples taken together. Clones with a SCNA score greater than the highest observed score for normal samples were considered copy number aberrant. For p018, p030 and p033, all clones except the one with the lowest SCNA score was considered copy number aberrant. (B) UMAPs of epithelial cells, color-coded by tissue sample type, cluster type, and copy number clone. Cluster type assignment is based on tissue origin and indicates if clusters are overrepresented in normal or tumor tissue samples (see also Supp. Fig. 3A-B). Quantification of epithelial cells, color-coded by cluster type or copy number clone, grouped by tissue sample type.

A

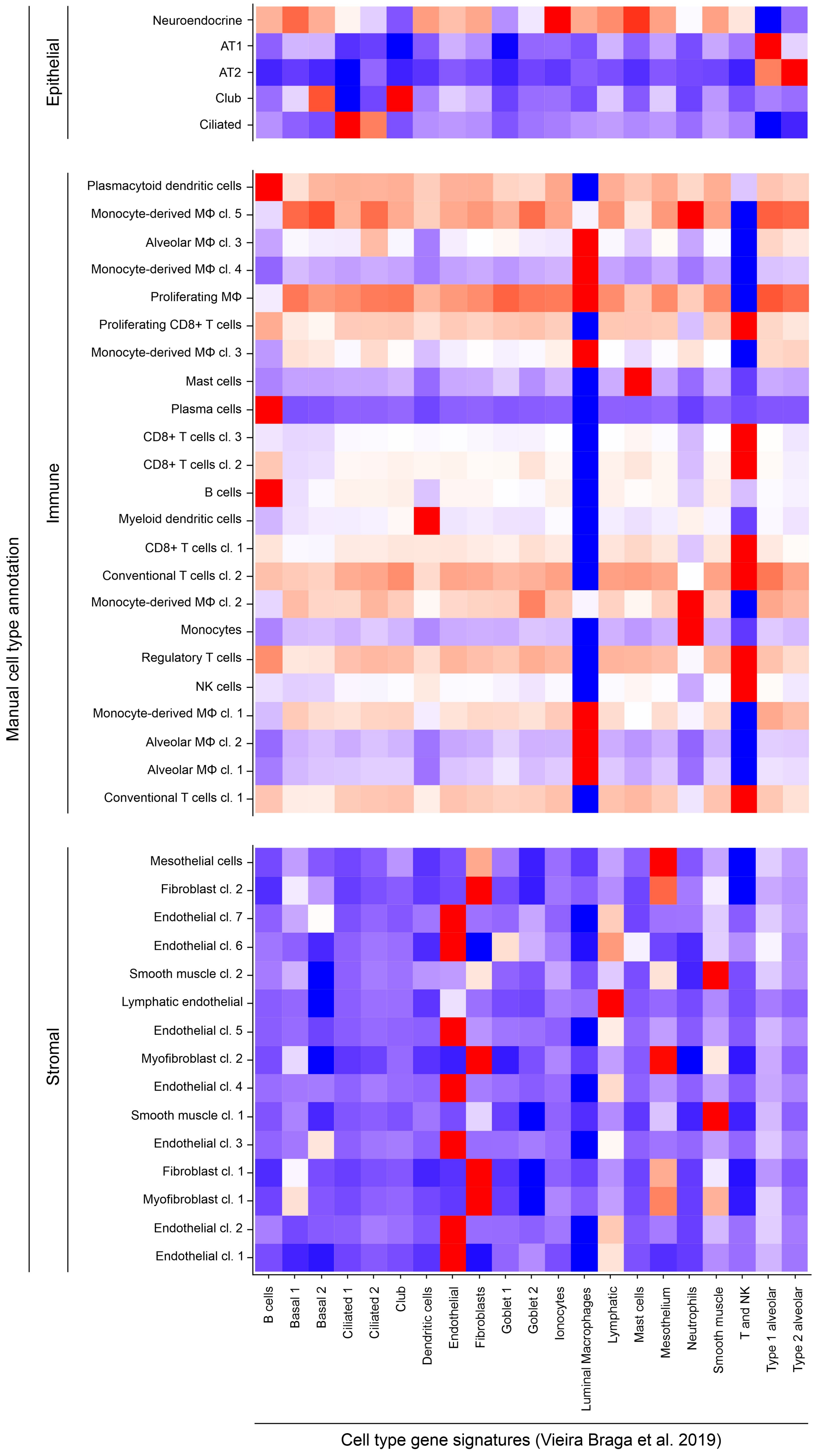

Epithelial
Neuroendocrine
AT1
AT2
Club
Ciliated
Immune
Plasmacytoid dendritic cells
Monocyte-derived MΦ cl. 5
Alveolar MΦ cl. 3
Monocyte-derived MΦ cl. 4
Proliferating MΦ
Proliferating CD8+ T cells
Monocyte-derived MΦ cl. 3
Mast cells
Plasma cells
CD8+ T cells cl. 3
CD8+ T cells cl. 2
B cells
Myeloid dendritic cells
CD8+ T cells cl. 1
Conventional T cells cl. 2
Monocyte-derived MΦ cl. 2
Monocytes
Regulatory T cells
NK cells
Monocyte-derived MΦ cl. 1
Alveolar MΦ cl. 2
Alveolar MΦ cl. 1
Conventional T cells cl. 1
Stromal
Mesothelial cells
Fibroblast cl. 2
Endothelial cl. 7
Endothelial cl. 6
Smooth muscle cl. 2
Lymphatic endothelial
Endothelial cl. 5
Myofibroblast cl. 2
Endothelial cl. 4
Smooth muscle cl. 1
Endothelial cl. 3
Fibroblast cl. 1
Myofibroblast cl. 1
Endothelial cl. 2
Endothelial cl. 1
Manual cell type annotation
B cells
Basal 1
Basal 2
Ciliated 1
Ciliated 2
Club
Dendritic cells
Endothelial
Fibroblasts
Goblet 1
Goblet 2
Ionocytes
Luminal Macrophages
Lymphatic
Mast cells
Mesothelium
Neutrophils
Smooth muscle
T and NK
Type 1 alveolar
Type 2 alveolar
Cell type gene signatures (Vieira Braga et al. 2019)

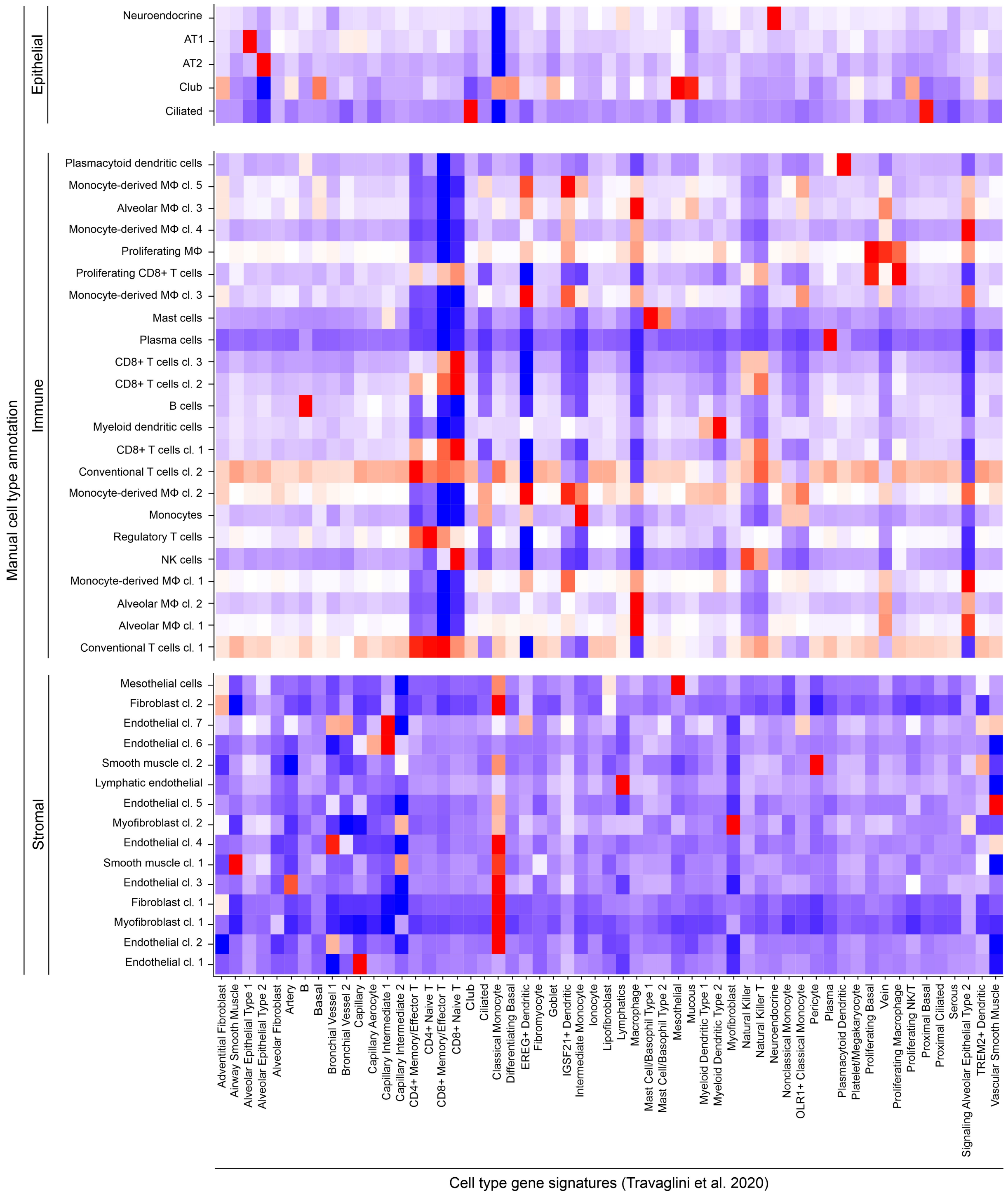

Supplemental Figure 5

(A+B) Mean module scores of cell type gene signatures according to (A) Vieira Braga et al. (doi: 10.1038/s41591-019-0468-5) or (B) Travaglini et al. (doi: 10.1038/s41586-020-2922-4) for manually annotated epithelial, immune and stromal cell clusters.

A

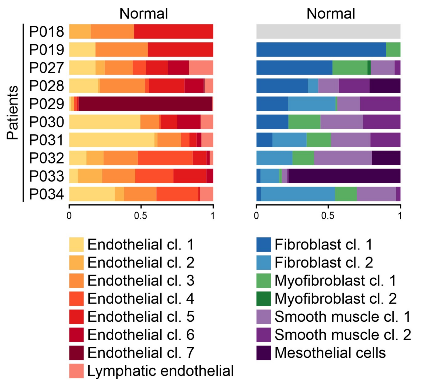

B

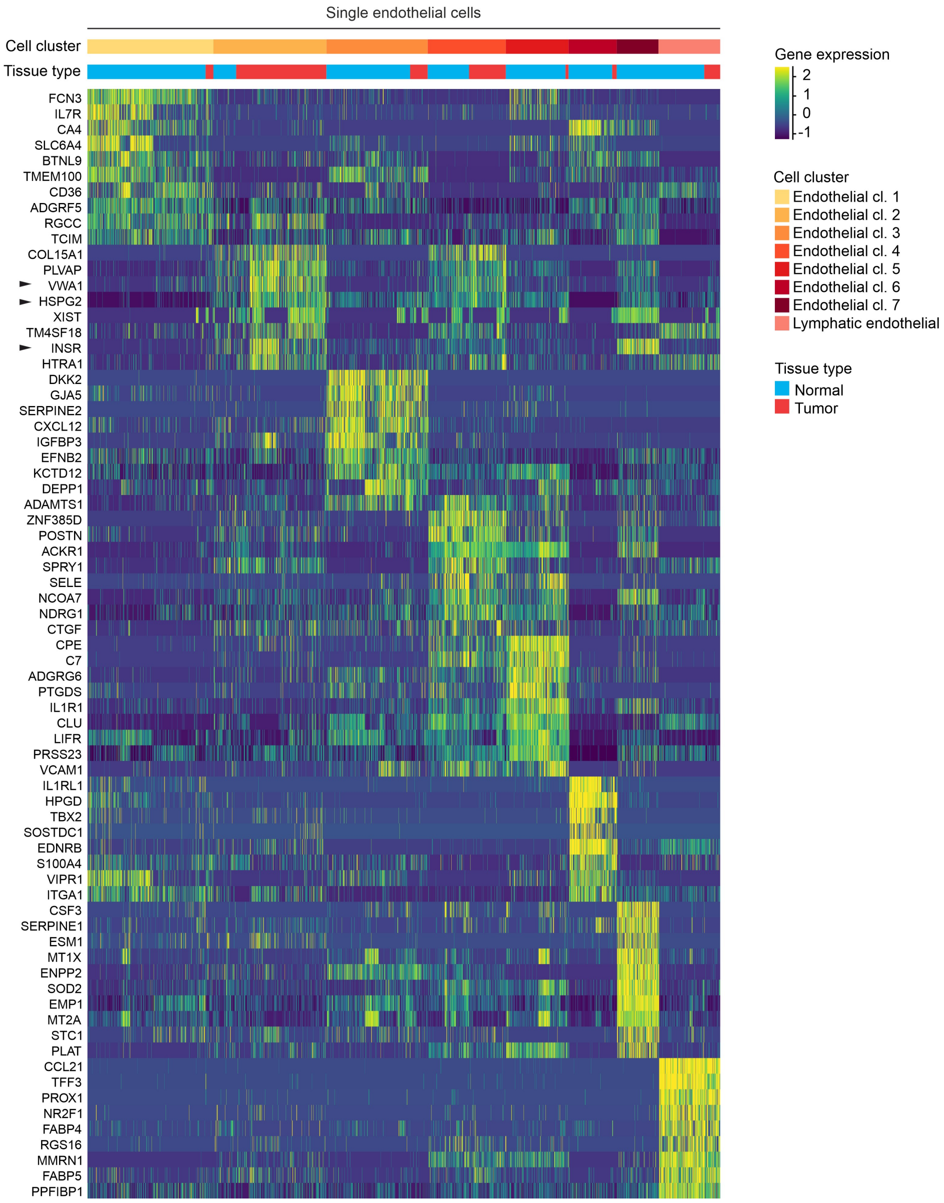

Supplemental Figure 6

(A) Quantification of endothelial and fibroblastic/muscle cell clusters per patient for normal tissue samples. (B) Differentially expressed genes of endothelial cell clusters, maximum top 10 genes showed per cluster, black arrowheads indicate genes mentioned in the main text.

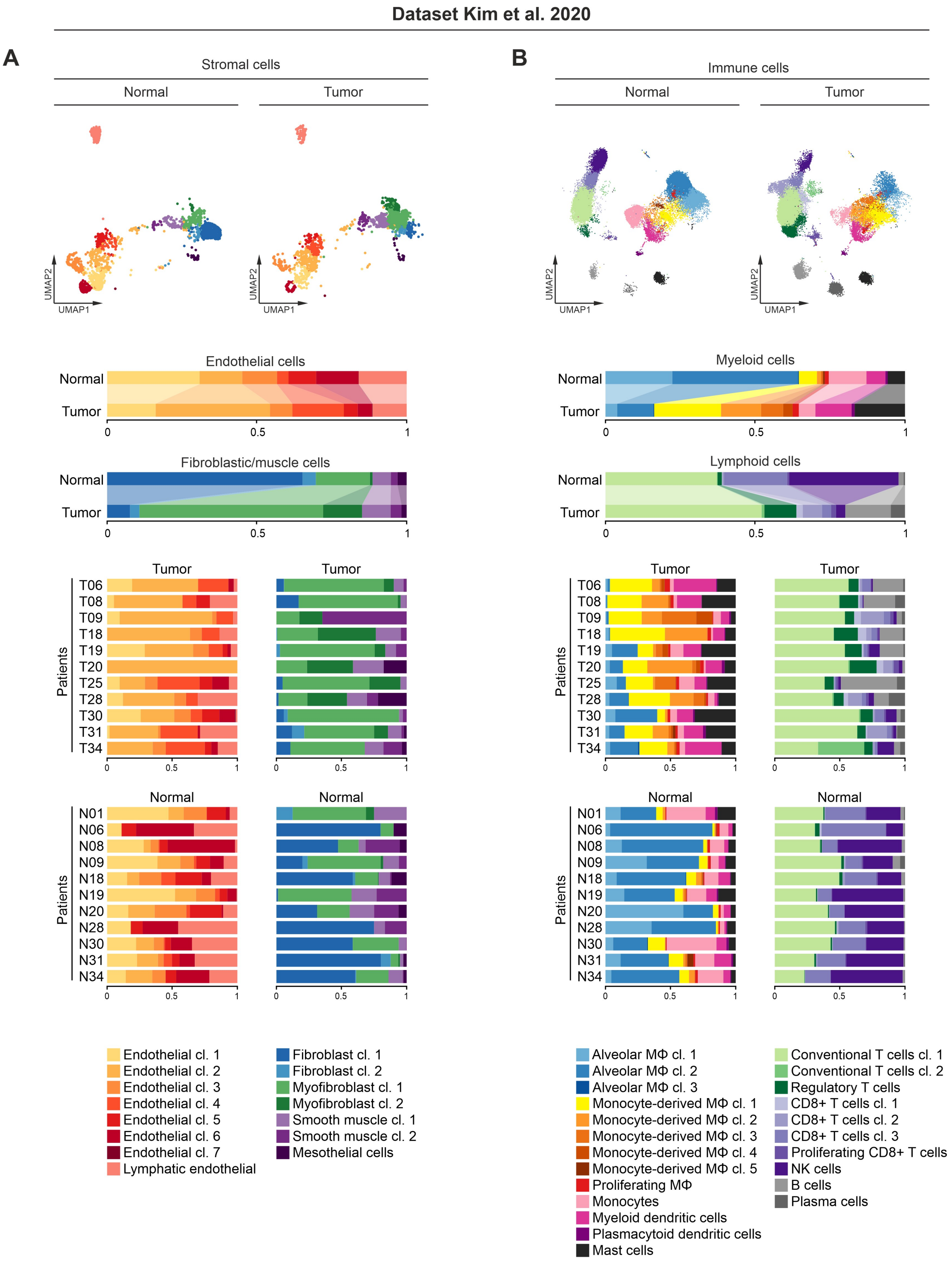

Dataset Kim et al. 2020
A
Stromal cells
Normal
Tumor
B
Immune cells
Normal
Tumor
UMAP1
UMAP2
Endothelial cells
Fibroblastic/muscle cells
Myeloid cells
Lymphoid cells
Patients
T06
T08
T09
T18
T19
T20
T25
T28
T30
T31
T34
N01
N06
N08
N09
N18
N19
N20
N28
N30
N31
N34
0
0.5
1
Endothelial cl. 1
Endothelial cl. 2
Endothelial cl. 3
Endothelial cl. 4
Endothelial cl. 5
Endothelial cl. 6
Endothelial cl. 7
Lymphatic endothelial
Fibroblast cl. 1
Fibroblast cl. 2
Myofibroblast cl. 1
Myofibroblast cl. 2
Smooth muscle cl. 1
Smooth muscle cl. 2
Mesothelial cells
Alveolar MΦ cl. 1
Alveolar MΦ cl. 2
Alveolar MΦ cl. 3
Monocyte-derived MΦ cl. 1
Monocyte-derived MΦ cl. 2
Monocyte-derived MΦ cl. 3
Monocyte-derived MΦ cl. 4
Monocyte-derived MΦ cl. 5
Proliferating MΦ
Monocytes
Myeloid dendritic cells
Plasmacytoid dendritic cells
Mast cells
Conventional T cells cl. 1
Conventional T cells cl. 2
Regulatory T cells
CD8+ T cells cl. 1
CD8+ T cells cl. 2
CD8+ T cells cl. 3
Proliferating CD8+ T cells
NK cells
B cells
Plasma cells

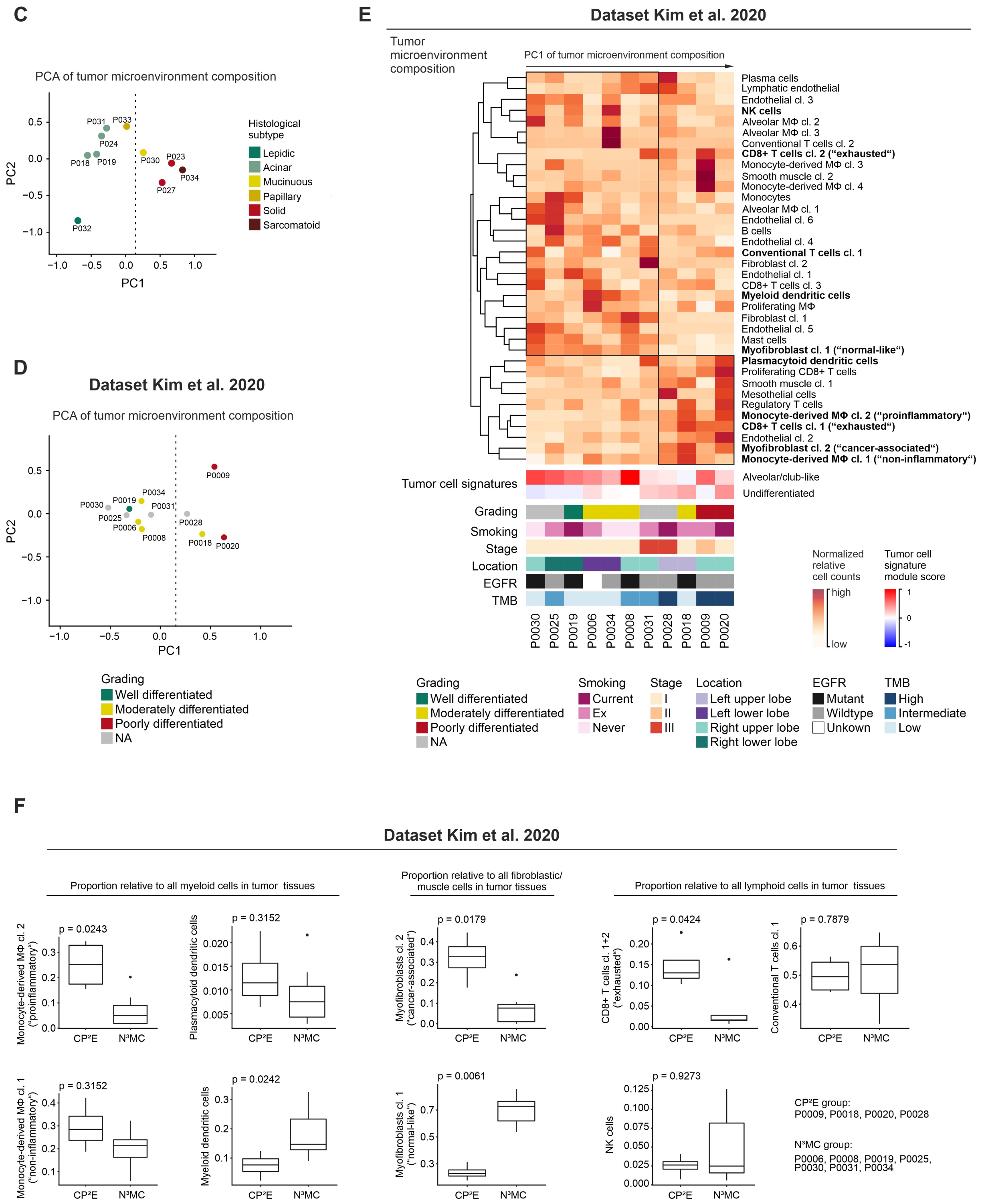

Supplemental Figure 7

(A-B, D-E) Gene expression and clinical data were obtained from Kim et al. 2020 (doi: 10.1038/s41467-020-16164-1) and preprocessed as our dataset. Single-cell transcriptomes were projected in the UMAP spaces of our dataset and cell type labels were transferred from our dataset using the “ingest” function of the “Scanpy” toolbox. (A-B) Projected UMAPs of all (A) stromal or (B) immune single-cell transriptomes of Kim et al., color-coded by cell cluster; and relative quantification of (A) endothelial and fibroblastic/muscle or (B) myeloid and lymphoid cell clusters per tissue type and patient.

(C-D) Principal component analysis of (C) our cohort or (D) the cohort of Kim et al. based on the proportion of stromal and immune cell clusters, color-coded by (C) histological subtype or (D) histological grade, patients indicated, dotted lines represent separation of patients in two main groups along principal component 1. (E) Normalized proportion of stromal and immune cell clusters and mean module scores of tumor cell signatures in the cohort of Kim et al., histopathological and clinical features indicated per patient (obtained from Kim et al. 2020, Fig. 2d), patients sorted along the first principal component from principal component analysis in (D), cell clusters included in the model in main Fig. 5H in bold. (F) Relative proportion of stromal and immune cell clusters in patient groups in the cohort of Kim et al.; n = 10, two-sided Mann-Whitney U test statistics.

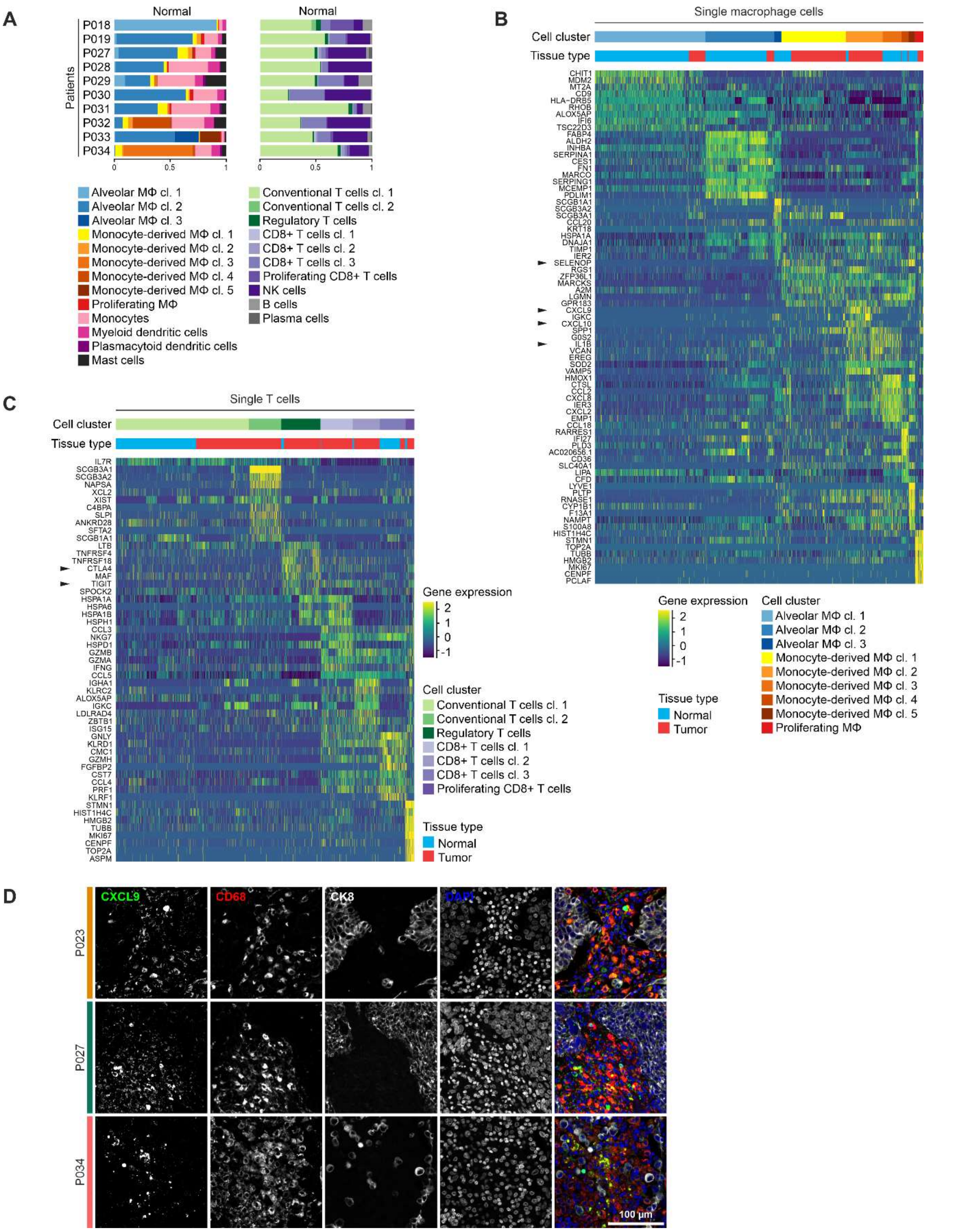

Supplemental Figure 8

(A) Quantification of myeloid and lymphoid cell clusters per patient for normal tissue samples. (B) Differentially expressed genes of macrophage cell clusters, maximum top 10 genes showed per cluster, black arrowheads indicate genes mentioned in the main text. (C) Differentially expressed genes of T cell clusters, maximum top 10 genes showed per cluster, black arrowheads indicate genes mentioned in the main text. (D) Multiplex immunofluorescence staining of proinflammatory macrophages using CXCL9 and CD68, and tumor cells using CK8, scale bar = 100 µm.

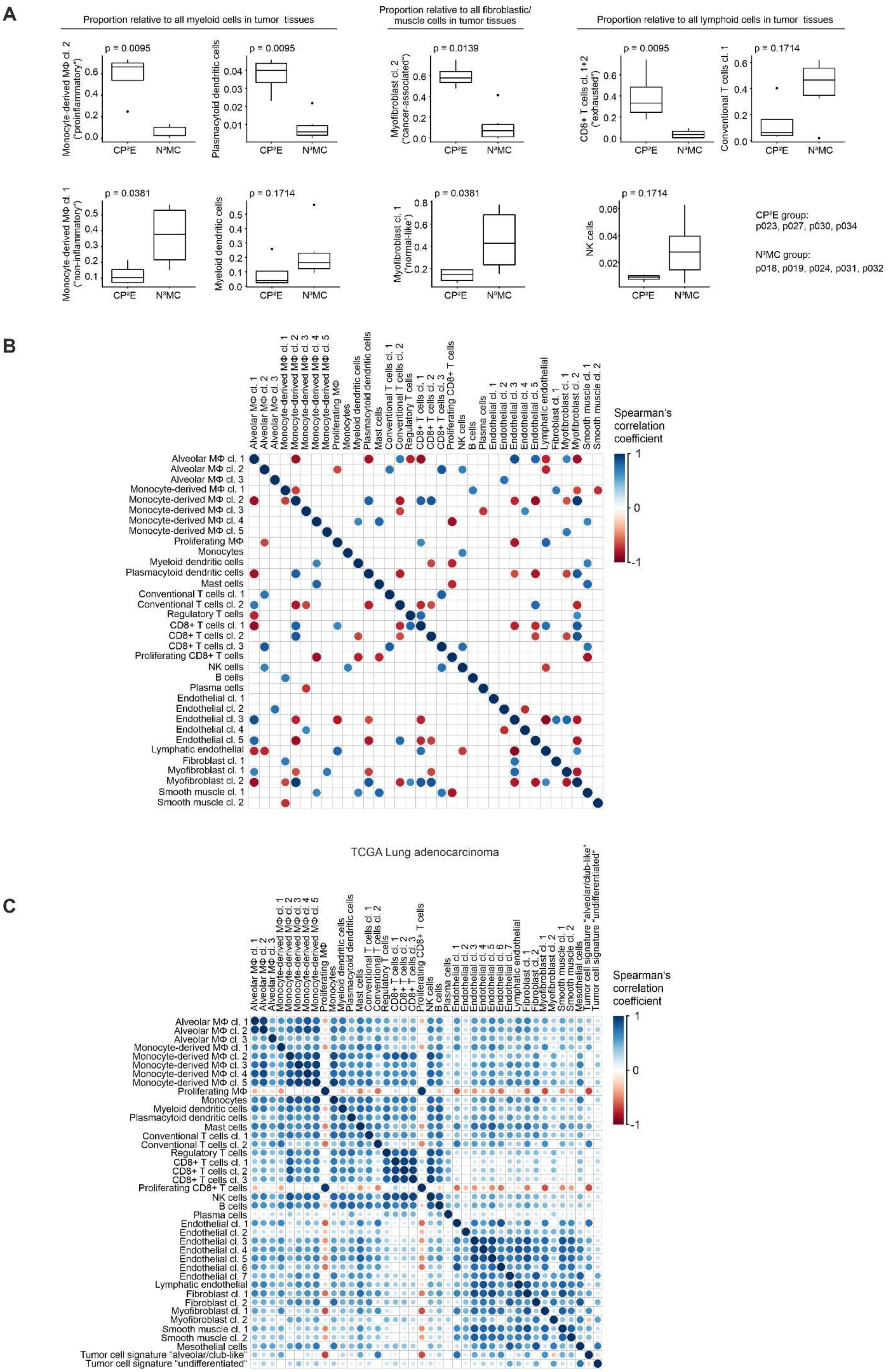
A
Proportion relative to all myeloid cells in tumor tissues
Proportion relative to all fibroblastic/ muscle cells in tumor tissues
Proportion relative to all lymphoid cells in tumor tissues
Monocyte-derived MΦ cl. 2 ("proinflammatory")
p = 0.0095
Plasmacytoid dendritic cells
p = 0.0095
Myofibroblast cl. 2 ("cancer-associated")
p = 0.0139
CD8+ T cells cl. 1+2 ("exhausted")
p = 0.0095
Conventional T cells cl. 1
p = 0.1714
Monocyte-derived MΦ cl. 1 ("non-inflammatory")
p = 0.0381
Myeloid dendritic cells
p = 0.1714
Myofibroblast cl. 1 ("normal-like")
p = 0.0381
NK cells
p = 0.1714
CP²E
N³MC
CP²E group:
p023, p027, p030, p034
N³MC group:
p018, p019, p024, p031, p032
B
Spearman's correlation coefficient
Alveolar MΦ cl. 1
Alveolar MΦ cl. 2
Alveolar MΦ cl. 3
Monocyte-derived MΦ cl. 1
Monocyte-derived MΦ cl. 2
Monocyte-derived MΦ cl. 3
Monocyte-derived MΦ cl. 4
Monocyte-derived MΦ cl. 5
Proliferating MΦ
Monocytes
Myeloid dendritic cells
Plasmacytoid dendritic cells
Mast cells
Conventional T cells cl. 1
Conventional T cells cl. 2
Regulatory T cells
CD8+ T cells cl. 1
CD8+ T cells cl. 2
CD8+ T cells cl. 3
Proliferating CD8+ T cells
NK cells
B cells
Plasma cells
Endothelial cl. 1
Endothelial cl. 2
Endothelial cl. 3
Endothelial cl. 4
Endothelial cl. 5
Lymphatic endothelial
Fibroblast cl. 1
Myofibroblast cl. 1
Myofibroblast cl. 2
Smooth muscle cl. 1
Smooth muscle cl. 2
TCGA Lung adenocarcinoma
C
Spearman's correlation coefficient
Endothelial cl. 6
Endothelial cl. 7
Fibroblast cl. 2
Mesothelial cells
Tumor cell signature "alveolar/club-like"
Tumor cell signature "undifferentiated"

Supplemental Figure 9

(A) Relative proportion of stromal and immune cell clusters in patient groups; n = 10, two-sided Mann-Whitney U test statistics. (B) Correlation of the proportion of immune and stromal cell clusters in tumor tissue samples, cell clusters occurring in less than three tumor tissue samples were excluded from analysis; Spearman's correlation statistics, only correlations with $p < 0.05$ shown. (C) Correlation of ssGSEA enrichment scores of cell cluster marker gene signatures in the TCGA lung adenocarcinoma cohort; Spearman's correlation statistics, only correlations with $p < 0.05$ shown.

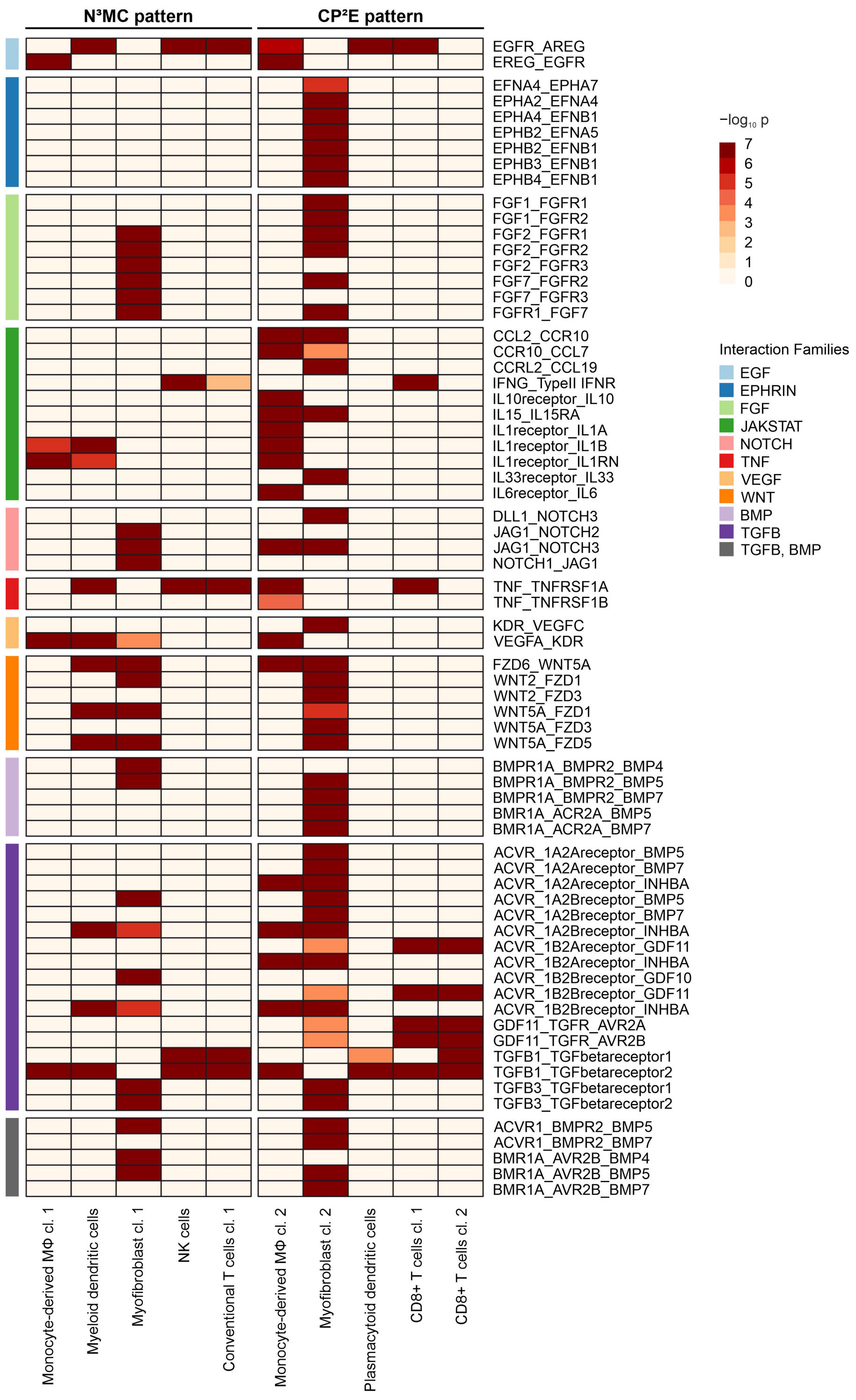

Supplemental Figure 10

Heatmap of significant interactions between tumor and microenvironmental cells as computed using the CellPhoneDB algorithm, filtered for interactions where receptors are expressed in tumor cells and ligands are expressed in microenvironmental cells of the N³MC or CP²E pattern, and filtered for high-confidence canonical receptors and ligands of relevant oncogenic pathways (see Supp. Table 6), color-coded by p-value.

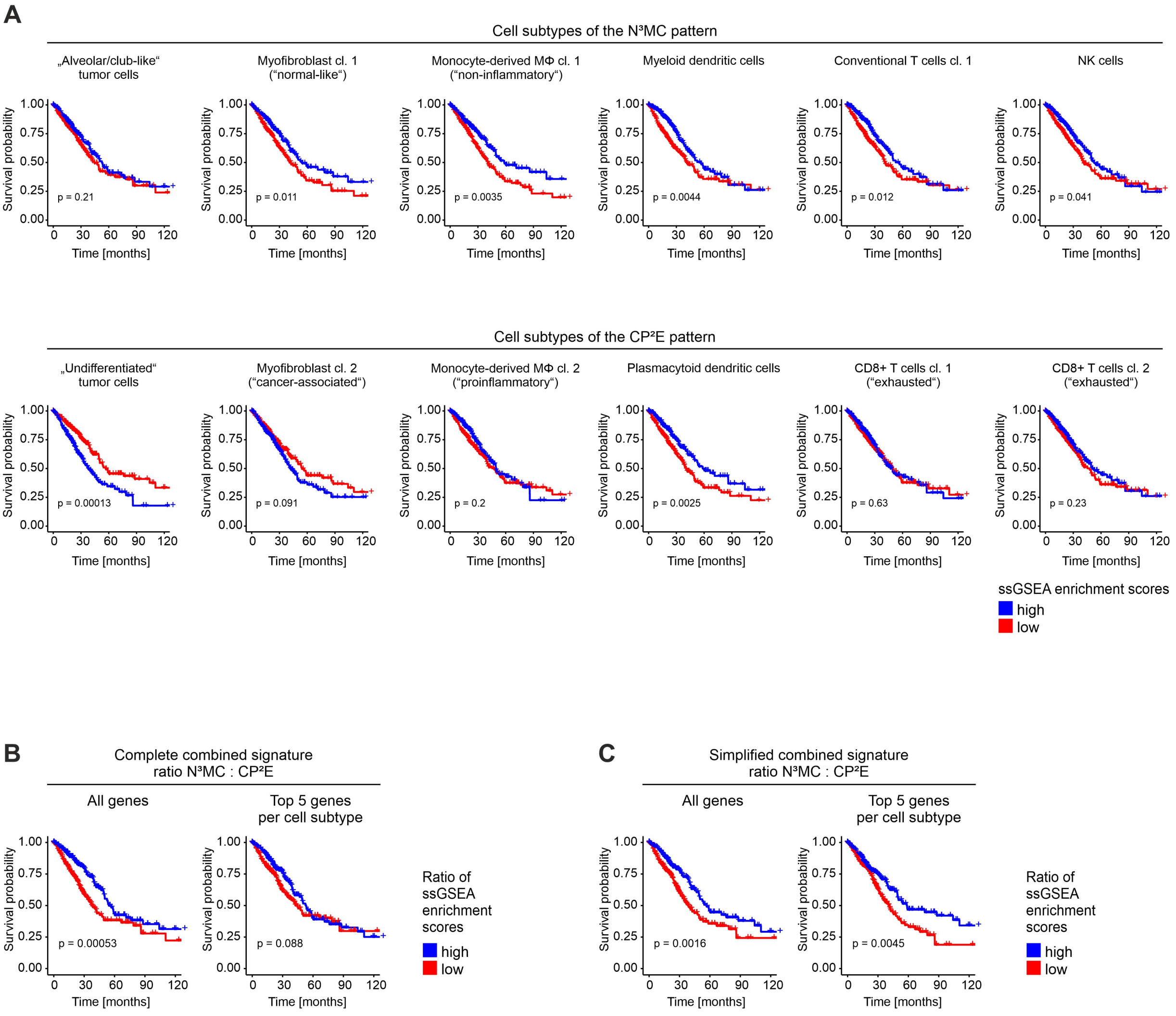

Supplemental Figure 11

(A-C) Kaplan-Meier overall survival curves of 524 cases from the TCGA lung adenocarcinoma cohort. (A) Cases were grouped by ssGSEA enrichment scores of marker gene sets of epithelial, immune and stromal cell subtypes which are characteristic for the N³MC and CP²E tumor microenvironmental patterns, for marker gene sets see Supp. Tables 7-10. (B) All marker genes or the top 5 marker genes per cell subtype of the N³MC and CP²E pattern (see (A)) were combined, respectively, and TCGA cases were grouped by the ratio of ssGSEA enrichment scores of the combined N³MC and CP²E gene sets. (C) All marker genes or the top 5 marker genes of myofibroblast cl. 1 and monocyte-derived macrophages cl. 1 (N³MC pattern, see (A)) or undifferentiated tumor cells and myofibroblast cl. 2 (CP²E pattern, see (A)) were combined, respectively, and TCGA cases were grouped by the ratio of ssGSEA enrichment scores of the simplified combined N³MC and CP²E gene sets. (A-C) “high” indicates ssGSEA enrichment score > median, “low” indicates ssGSEA enrichment score ≤ median, n = 524, log-rank statistics. (B-C) For complete and simplified short combined gene sets, see Supp. Table 11.
